# Supplementary material for: Integrating protein networks and machine learning for disease stratification in the Hereditary Spastic Paraplegias
Source: iScience. 2021 Apr 28;24(5):102484. doi: 10.1016/j.isci.2021.102484 (PMC8169945; doi:10.1016/j.isci.2021.102484)
Supplement: Document S1. Figures S1–S11 and Tables S1, S2, and S5–S7 [file mmc1.pdf]

iScience, Volume *24*

Supplemental information

Integrating protein networks and machine learning for disease stratification in the Hereditary Spastic Paraplegias

Nikoleta Vavouraki, James E. Tomkins, Eleanna Kara, Henry Houlden, John Hardy, Marcus J. Tindall, Patrick A. Lewis, and Claudia Manzoni

Supplementary Figures

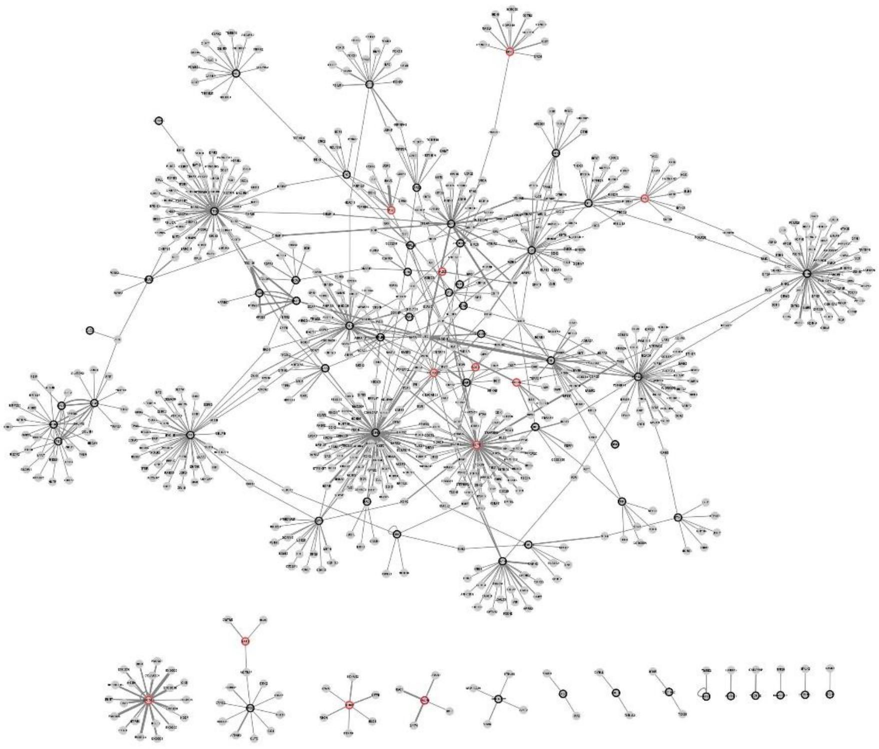

Figure S1. The global HSP-PPIN, related to Figure 1
The global HSP-PPIN is a visualisation of all binary interactions of the HSP seeds and test seeds that were collected through the online tool PINOT following filtering based on the final score. The nodes corresponding to the HSP seeds have a black border, while the test seeds have a red border. The thickness of each edge positively correlates with the final score of the respective interaction as calculated by PINOT, which acts as a proxy for interaction confidence.

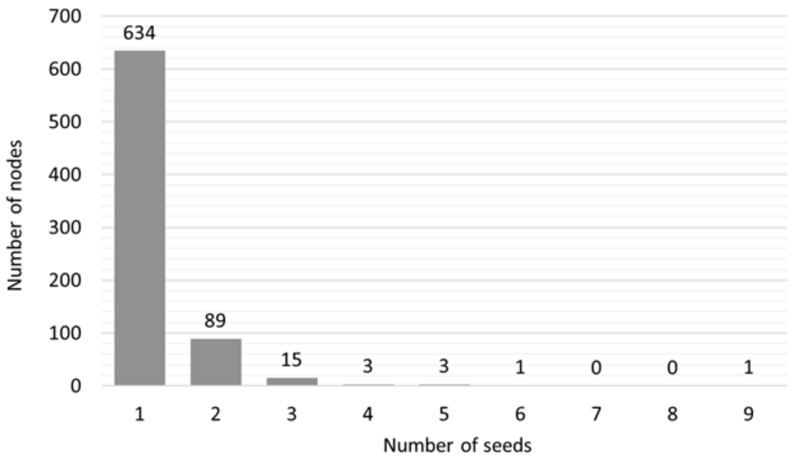

Figure S2. Node degree distribution of the proteins of the global HSP-PPIN based on their connectivity with seeds, related to Figure 1
The nodes of the global HSP-PPIN were analysed to calculate the number of seeds to which they connect. The interactors connected to one seed (n=634) were not included in further analyses.

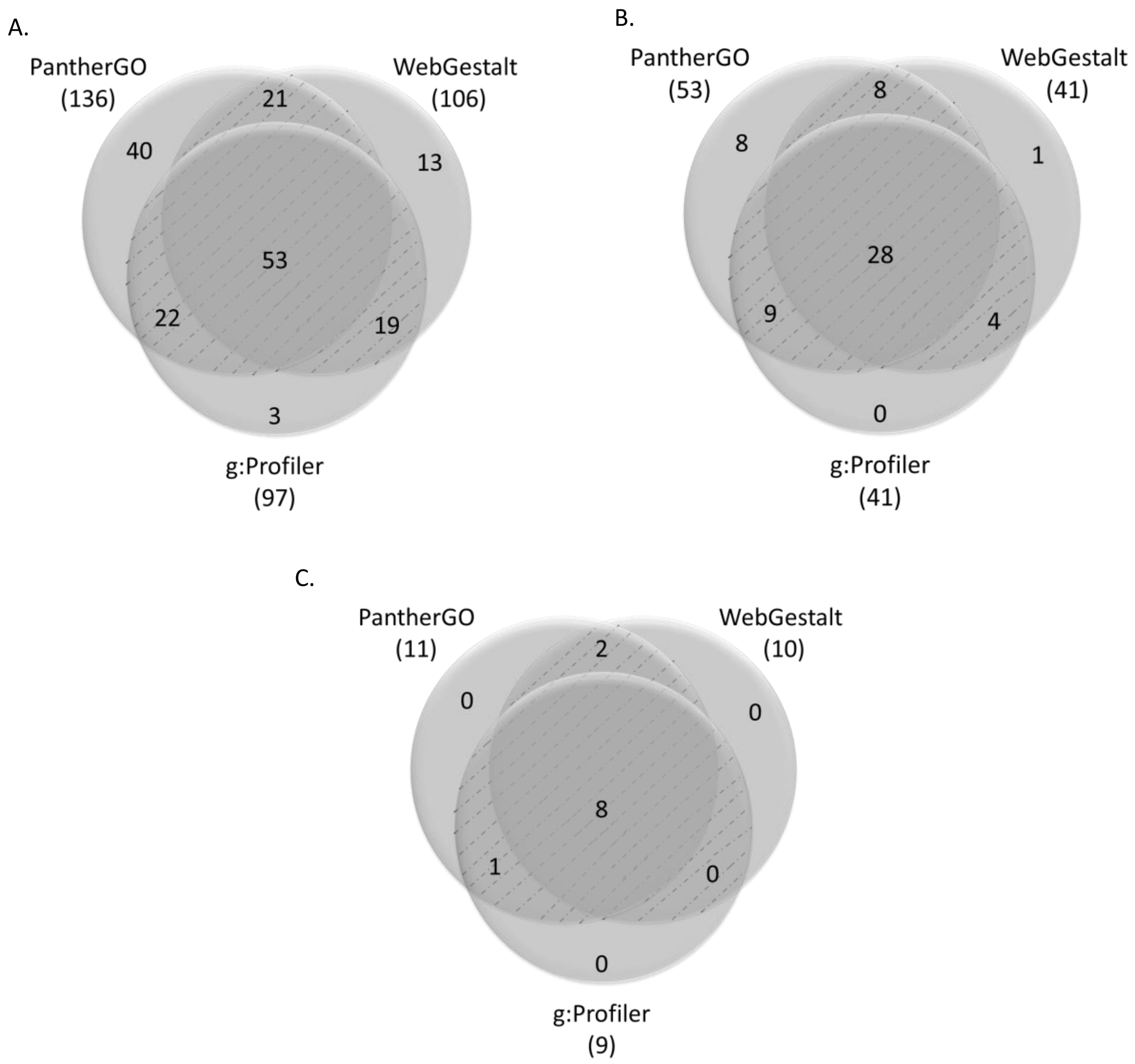

Figure S3. Overlap of the three functional enrichment tools for the analysis of the core HSP-PPIN, related to Figure 2 and 3

The results from the functional enrichment analysis of the core HSP-PPIN were compared across the functional enrichment tools used, in three levels: single GO-BP terms (n=171) (**A**), semantic classes (n=58) (**B**), and functional blocks (n=11) (**C**).

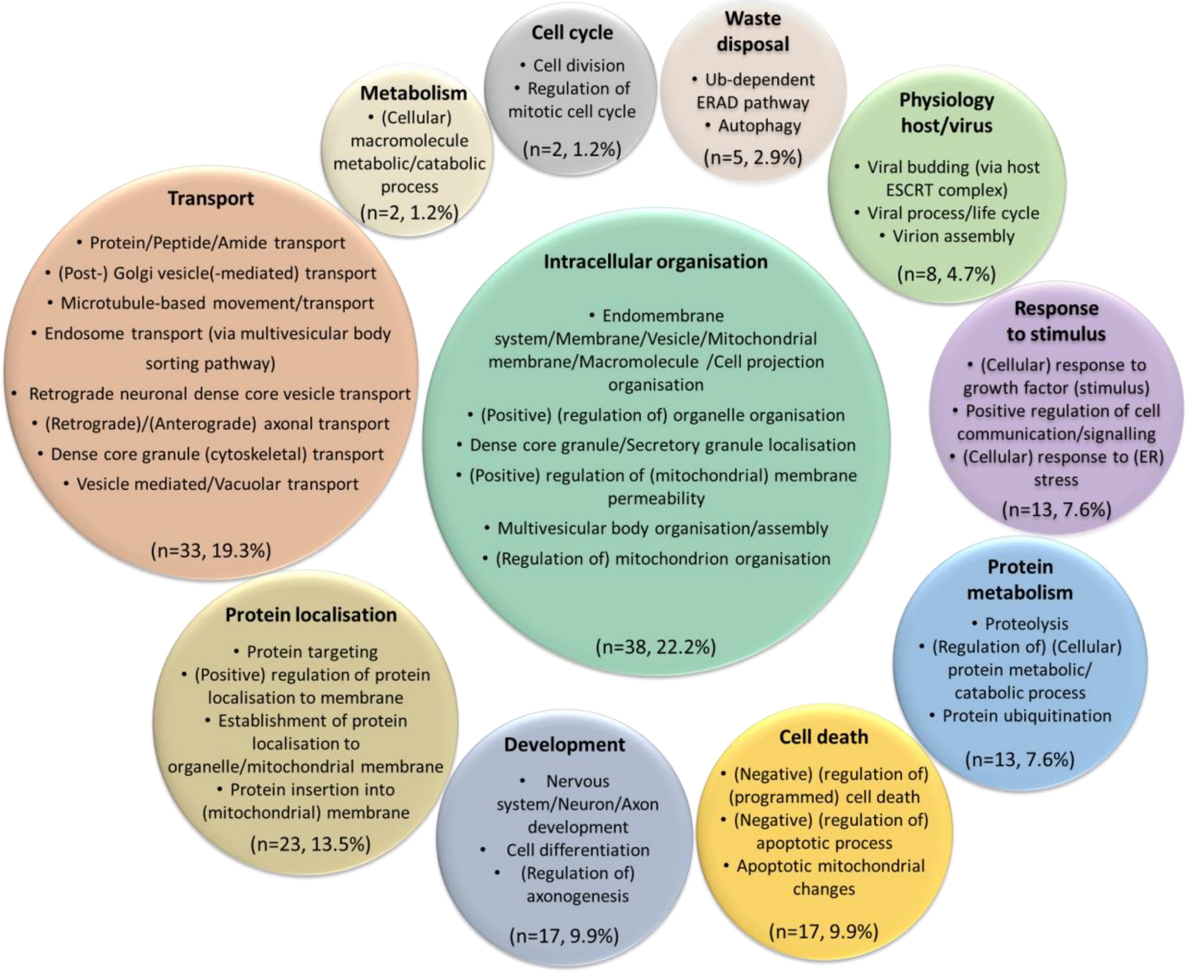

Figure S4. Detailed graphical representation of the functional enrichment of the core HSP-PPIN, related to Figure 3

Functional enrichment was performed for the components of the core HSP-PPIN. The resulted GO-BP terms (n=171) (see Table S3) were grouped into semantic classes and then into functional blocks (name of each circle, bolded). The number and percentage of terms in each functional block was calculated by merging the data from g:Profiler, WebGestalt, and PantherGO as described in STAR Methods. Examples of GO-BP terms are included inside the circle of each functional block.

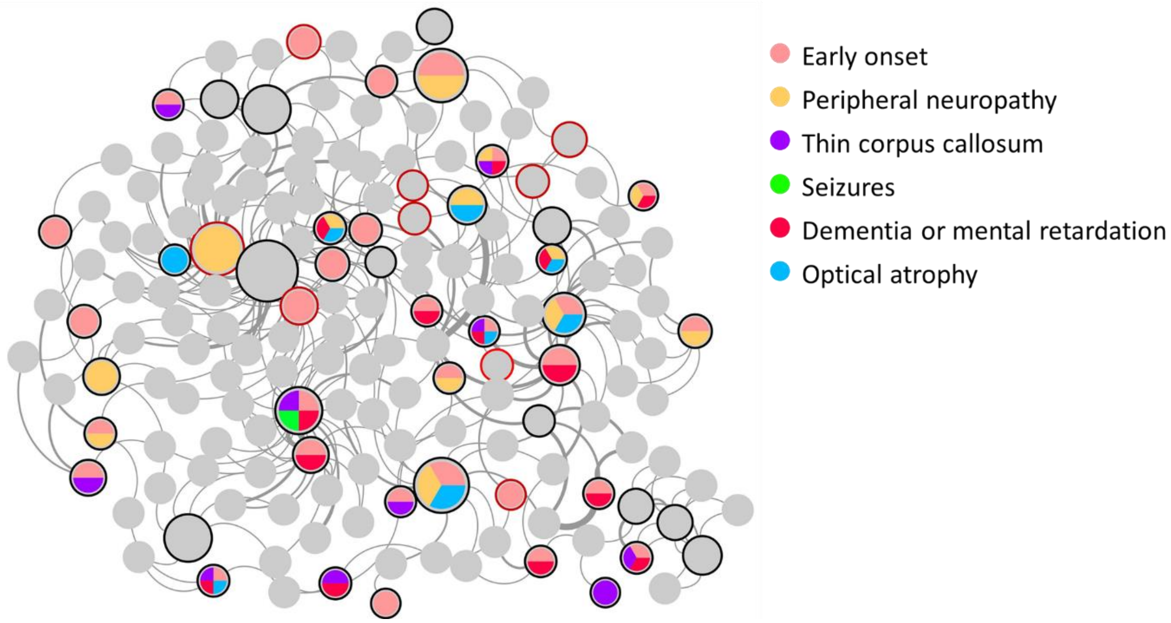

Figure S5. Visualisation of clinical characteristics of HSPs caused by each HSP gene of the core HSP-PPIN, related to Figure 4

The presence of clinical characteristics in HSPs is visualised in the core HSP-PPIN through the colour of each node. The colour-function correspondence is located on the top right of the image, while the grey nodes did not present any of the clinical features. The nodes corresponding to the HSP seeds have a black border, while the test seeds have a red border. The size of each node correlates with its degree. The thickness of each edge correlates with its final score as calculated by PINOT.

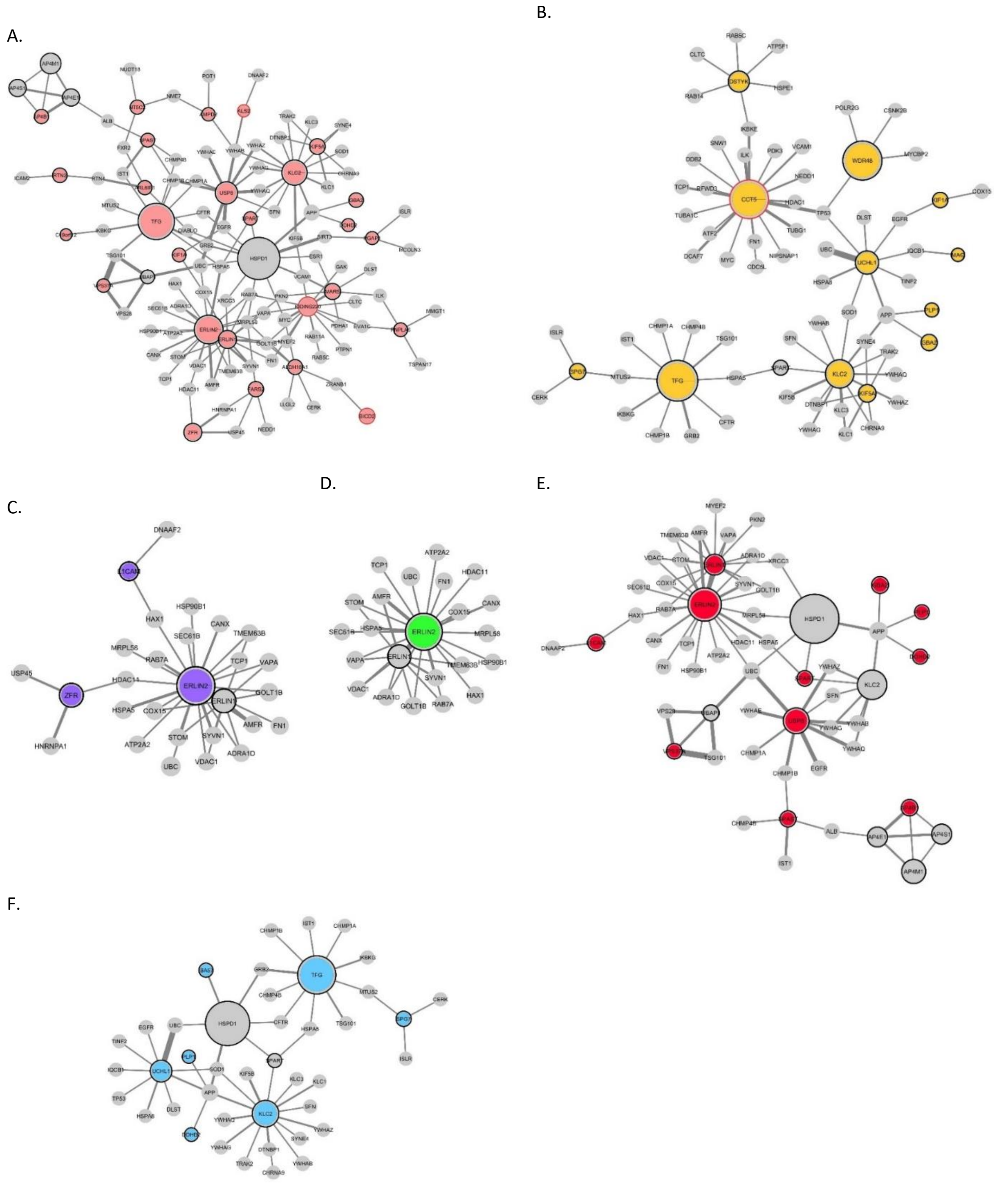

Figure S6. Comparison of the core network of seeds with the analysed clinical features, related to Figure 4

The presence of clinical characteristics in HSPs is visualised in the core networks by the colour of each node for early onset (**A**), peripheral neuropathy (**B**), thin corpus callosum (**C**), seizures, (**D**), dementia or mental retardation (**E**), and optic atrophy (**F**), while the nodes without that clinical feature are grey. The nodes corresponding to the HSP seeds have a black border, while the test seeds have a red border. The size of each node correlates with its degree. The thickness of each edge correlates with its final score as calculated by PINOT.

A.

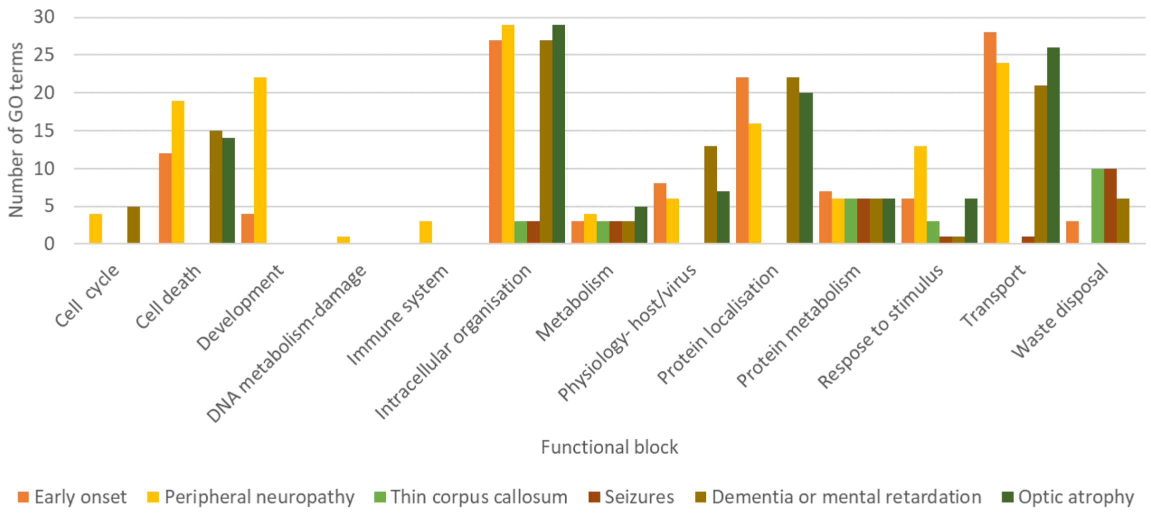

B.

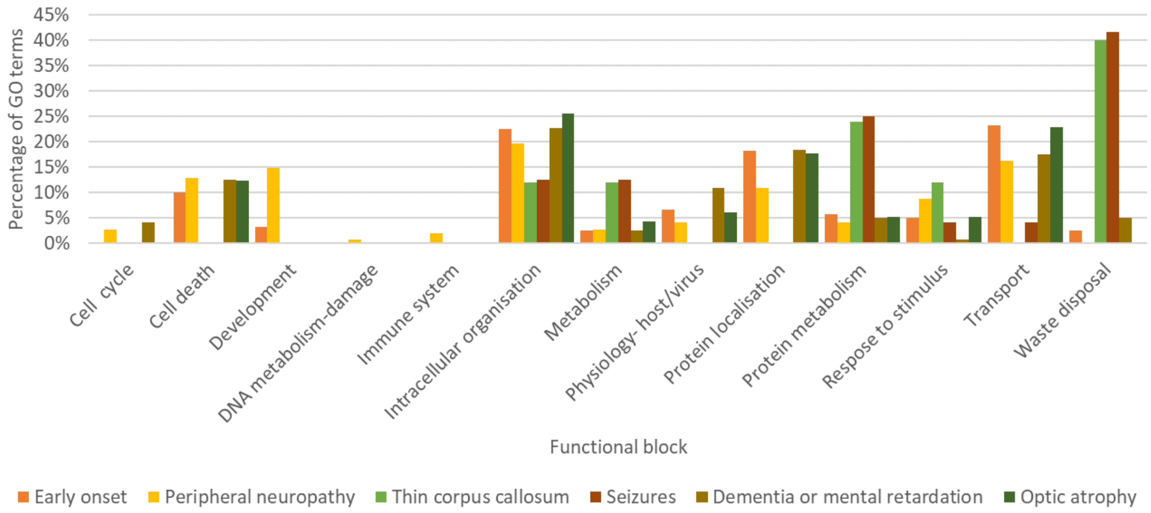

Figure S7. Functional enrichment of the core HSP-PPIN for each group related to a clinical characteristic, related to Figure 4

The number (**A**) and percentage (**B**) of the GO-BP terms in each functional block is shown for all six groups of genes related to different clinical features. The results are calculated from the functional enrichment data of g:Profiler, PantherGO, and WebGestalt (raw data in Table S4).

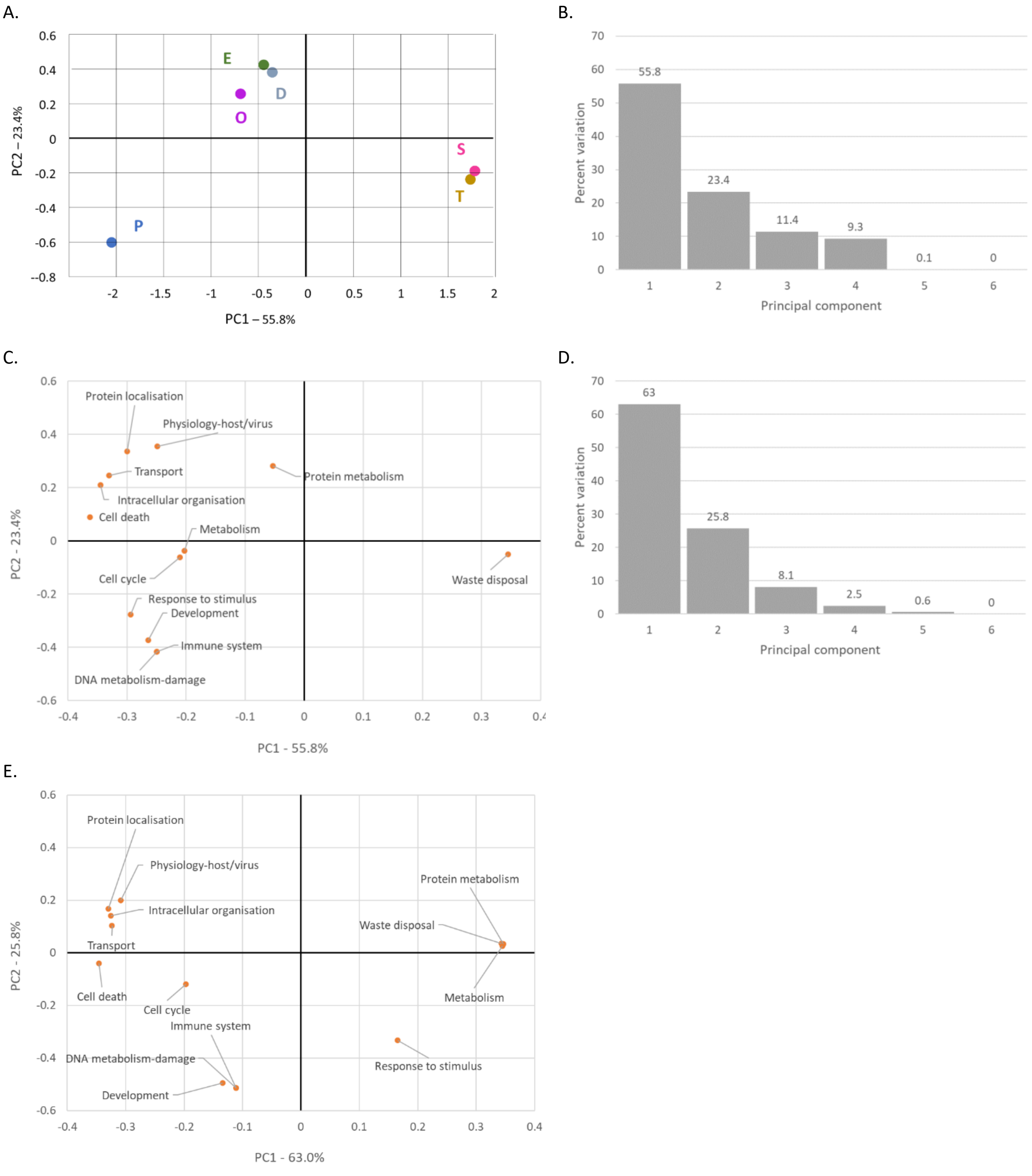

Figure S8. Comparison of the functional profiles of the 6 subdivisions of the core HSP-PPIN based on clinical features using Principal Component Analysis, related to Figure 4

The number (**A**, **B**, **C**) and percentage (**D**, **E**) of GO-BP terms for each functional block were analysed with PCA through R. (**A**) The PCA graph is showing the distribution of the gene groups in the PC1 and PC2 axes. The axes have been adjusted based on their respective explained variation. (**B**, **D**) The scree plot is showing the explained variation of the data for PC1 to PC6. (**C**, **E**) The loading scores of each variable (here functional blocks) are plotted against PC1 and PC2, indicating which functions drive the localisation of the gene groups in the PCA graph. E: Early onset, P: Peripheral neuropathy, T: Thin corpus callosum, S: Seizures, D: Dementia or mental retardation, O: Optic atrophy

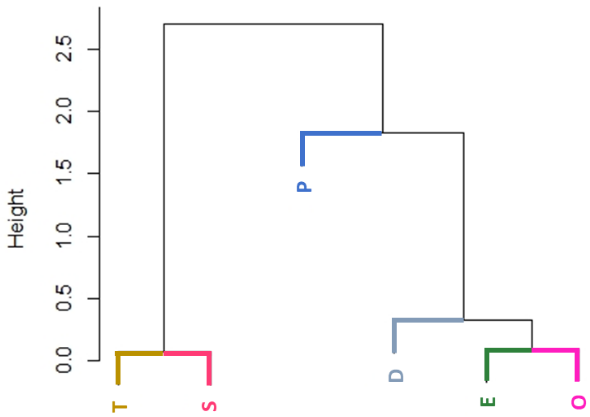

Figure S9. Cluster dendrogram for the number of GO-BP terms in the enrichment of the clinical subnetworks following PCA, related to Figure 4

Cluster dendrogram produced based on hierarchical clustering of the gene groups as analysed in Figure 4. E: Early onset, P: Peripheral neuropathy, T: Thin corpus callosum, S: Seizures, D: Dementia or mental retardation, O: Optic atrophy

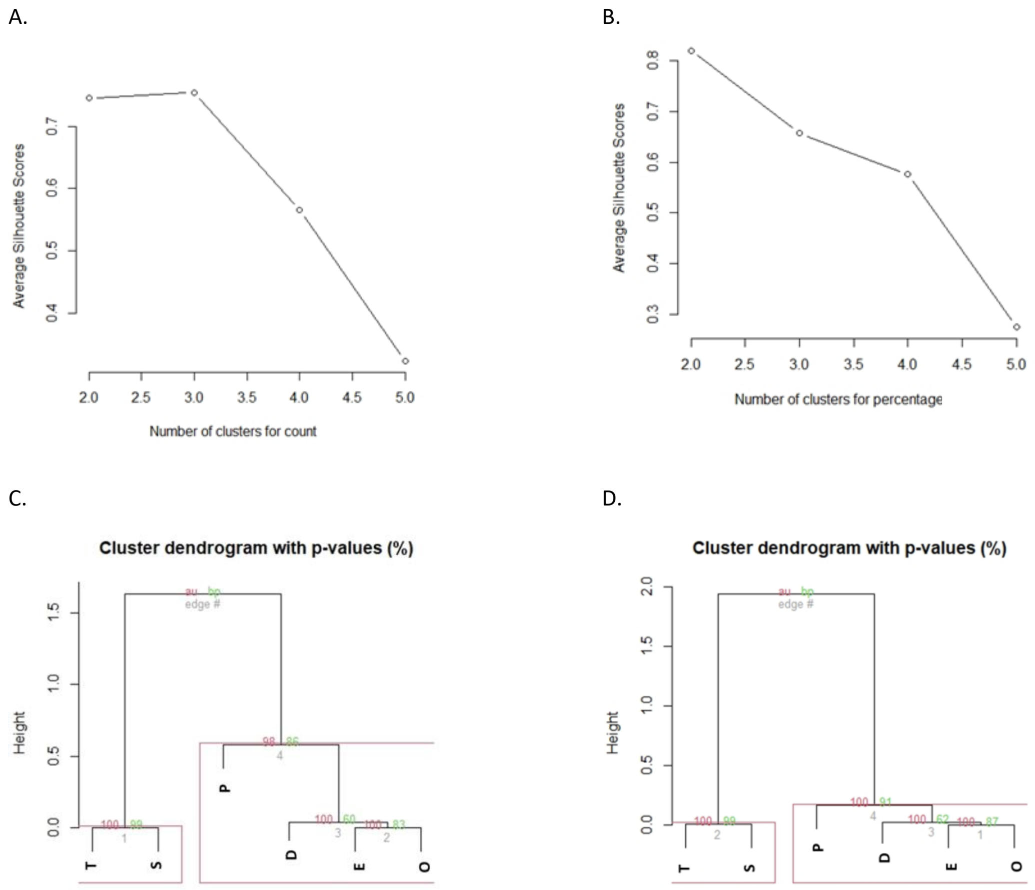

Figure S10. Evaluating the optimal number of clusters using the Silhouette method and the Multiscale bootstrap resampling through R, related to Figure 4

The analysis was based on the number (**A**, **C**) and percentage (**B**, **D**) of the GO-BP terms in functional block. The graphs based on the Silhouette method (**A**, **B**) show the optimal number of clusters being the one with the highest value, whereas based on multiscale bootstrap resampling (**C**, **D**) the recommended clusters are framed in red boxes. E: Early onset, P: Peripheral neuropathy, T: Thin corpus callosum, S: Seizures, D: Dementia or mental retardation, O: Optic atrophy

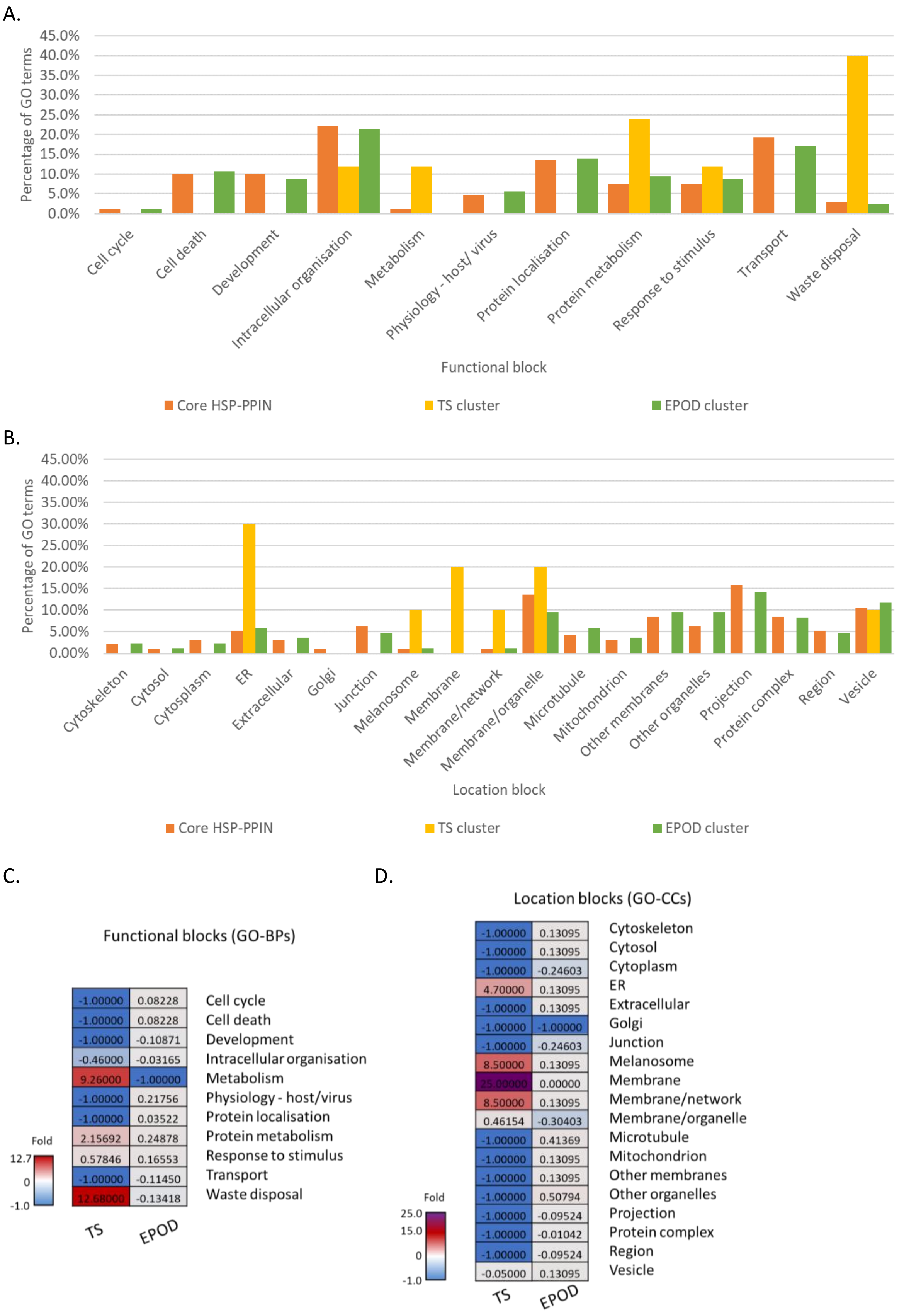
A.
Percentage of GO terms
45.0%
40.0%
35.0%
30.0%
25.0%
20.0%
15.0%
10.0%
5.0%
0.0%
Cell cycle
Cell death
Development
Intracellular organisation
Metabolism
Physiology - host/ virus
Protein localisation
Protein metabolism
Response to stimulus
Transport
Waste disposal
Functional block
Core HSP-PPIN
TS cluster
EPOD cluster
B.
Percentage of GO terms
45.00%
40.00%
35.00%
30.00%
25.00%
20.00%
15.00%
10.00%
5.00%
0.00%
Cytoskeleton
Cytosol
Cytosplasm
ER
Extracellular
Golgi
Junction
Melanosome
Membrane
Membrane/network
Membrane/organelle
Microtubule
Mitochondrion
Other membranes
Other organelles
Projection
Protein complex
Region
Vesicle
Location block
Core HSP-PPIN
TS cluster
EPOD cluster
C.
Functional blocks (GO-BPs)
-1.00000 0.08228 Cell cycle
-1.00000 0.08228 Cell death
-1.00000 -0.10871 Development
-0.46000 -0.03165 Intracellular organisation
9.26000 -1.00000 Metabolism
-1.00000 0.21756 Physiology - host/virus
-1.00000 0.03522 Protein localisation
2.15692 0.24878 Protein metabolism
0.57846 0.16553 Response to stimulus
-1.00000 -0.11450 Transport
12.68000 -0.13418 Waste disposal
Fold
12.7
0
-1.0
TS
EPOD
D.
Location blocks (GO-CCs)
-1.00000 0.13095 Cytoskeleton
-1.00000 0.13095 Cytosol
-1.00000 -0.24603 Cytoplasm
4.70000 0.13095 ER
-1.00000 0.13095 Extracellular
-1.00000 -1.00000 Golgi
-1.00000 -0.24603 Junction
8.50000 0.13095 Melanosome
25.00000 0.00000 Membrane
8.50000 0.13095 Membrane/network
0.46154 -0.30403 Membrane/organelle
-1.00000 0.41369 Microtubule
-1.00000 0.13095 Mitochondrion
-1.00000 0.13095 Other membranes
-1.00000 0.50794 Other organelles
-1.00000 -0.09524 Projection
-1.00000 -0.01042 Protein complex
-1.00000 -0.09524 Region
-0.05000 0.13095 Vesicle
Fold
25.0
15.0
0
-1.0
TS
EPOD

Figure S11. Comparison of the enrichment profile of the two clusters of the core HSP-PPIN, related to Figure 5

The analysis is based on the percentage of GO terms in each functional or location block as resulted from the enrichment of GO-BP terms (**A**, **C**) and GO-CC terms (**B**, **D**). The comparison is expressed as a fold change compared to the profile of the core HSP-PPIN (**C**, **D**).

Supplementary Tables

Table S1. Sources of variation in the clinical phenotype of HSP patients, related to Figure 1	
**Age of onset**	Early childhood up to late adulthood
**Form**	Pure and complex
**Mode of inheritance**	Autosomal recessive, autosomal dominant, X-linked, mitochondrial and unknown
**Usual Symptoms**	Bilateral spasticity and weakness of the lower body, leg hypertonicity, positive Babinski sign, muscle weakness, hyperreflexia, bladder dysfunction, loss of vibration sensation in the ankles, and pes cavus
**Additional symptoms present in some complex forms**	Cerebellar ataxia, epilepsy, cognitive or mental impairment, cataracts, retinal alteration, optic atrophy, peripheral neuropathy, dystonia, and parkinsonism

Table S2. The gene (or locus) and protein name responsible for each HSP type, related to Figure 1			
**Gene name (or locus)**	**Protein name (previous name)**	**UniProt identifier (SwissProt)**	**HSP type**
L1CAM	neural cell adhesion molecule L1	P32004	SPG1
PLP1	myelin proteolipid protein	P60201	SPG2
ATL1	atlastin-1	Q8WXF7	SPG3A
SPAST	spastin	Q9UBP0	SPG4
CYP7B1	25-hydroxycholesterol 7-alpha-hydroxylase	O75881	SPG5A
NIPA1	magnesium transporter NIPA1	Q7RTP0	SPG6
SPG7	paraplegin	Q9UQ90	SPG7
WASHC5	WASH complex subunit 5 (strumpellin)	Q12768	SPG8
ALDH18A1	delta-1-pyrroline-5-carboxylate synthase	P54886	SPG9A/SPG9B
KIF5A	kinesin heavy chain isoform 5A	Q12840	SPG10
SPG11	spatacsin	Q96JI7	SPG11
RTN2	reticulon-2	O75298	SPG12
HSPD1	60 kDa heat shock protein, mitochondrial	P10809	SPG13
(3q27-q28)			SPG14
ZFYVE26	zinc finger FYVE domain-containing protein 26 (spastizin)	Q68DK2	SPG15
(Xq11.2)			SPG16
BSCL2	seipin	Q96G97	SPG17
ERLIN2	erlin-2	O94905	SPG18/SPG37
(9q33-q34)			SPG19
SPART	spartin	Q8N0X7	SPG20
SPG21	maspardin	Q9NZD8	SPG21
SLC16A2	monocarboxylate transporter 8	P36021	SPG22
DSTYK	dual serine/threonine and tyrosine protein kinase	Q6XUX3	SPG23
(13q14)			SPG24
(6q23-24.1)			SPG25
B4GALNT1	beta-1,4 N-acetylgalactosaminyltransferase 1	Q00973	SPG26
(10q22.1-q24.1)			SPG27
DDHD1	phospholipase DDHD1	Q8NEL9	SPG28
(1p31.1-p21.1)			SPG29
KIF1A	kinesin-like protein KIF1A	Q12756	SPG30
REEP1	receptor expression-enhancing protein 1	Q9H902	SPG31
(14q12-q21)			SPG32

Table S2. (continued) The gene (or locus) and protein name responsible for each HSP type, related to Figure 1			
**Gene name (or locus)**	**Protein name (previous name)**	**UniProt identifier (SwissProt)**	**HSP type**
ZFYVE27	protrudin	Q5T4F4	SPG33
(Xq24-q25)			SPG34
FA2H	fatty acid 2-hydroxylase	Q7L5A8	SPG35
(12q23-q24)			SPG36
(4p16-p15)			SPG38
PNPLA6	neuropathy target esterase	Q8IY17	SPG39
(11p14.1-p11.2)			SPG41
SLC33A1	acetyl-coenzyme A transporter 1	O00400	SPG42
C19orf12	protein C19orf12	Q9NSK7	SPG43
GJC2	gap junction gamma-2 protein	Q5T442	SPG44
NT5C2	cytosolic purine 5'-nucleotidase	P49902	SPG45/SPG65
GBA2	non-lysosomal glucosylceramidase	Q9HCG7	SPG46
AP4B1	AP-4 complex subunit beta-1	Q9Y6B7	SPG47
AP5Z1	AP-5 complex subunit zeta-1	O43299	SPG48
TECPR2	tectonin beta-propeller repeat-containing protein 2	O15040	SPG49
AP4M1	AP-4 complex subunit mu-1	O00189	SPG50
AP4E1	AP-4 complex subunit epsilon-1	Q9UPM8	SPG51
AP4S1	AP-4 complex subunit sigma-1	Q9Y587	SPG52
VPS37A	vacuolar protein sorting-associated protein 37A	Q8NEZ2	SPG53
DDHD2	phospholipase DDHD2	O94830	SPG54
C12orf65	probable peptide chain release factor C12orf65, mitochondrial	Q9H3J6	SPG55
CYP2U1	cytochrome P450 2U1	Q7Z449	SPG56
TFG	protein TFG	Q92734	SPG57
KIF1C	kinesin-like protein KIF1C	O43896	SPG58
USP8	ubiquitin carboxyl-terminal hydrolase 8	P40818	SPG59
WDR48	WD repeat-containing protein 48	Q8TAF3	SPG60

**Table S2. (continued) The gene (or locus) and protein name responsible for each HSP type, related to Figure 1**			
**Gene name (or locus)**	**Protein name (previous name)**	**UniProt identifier (SwissProt)**	**HSP type**
ARL6IP1	ADP-ribosylation factor-like protein 6-interacting protein 1	Q15041	SPG61
ERLIN1	erlin-1	O75477	SPG62
AMPD2	AMP deaminase 2	Q01433	SPG63
ENTPD1	ectonucleoside triphosphate diphosphohydrolase 1	P49961	SPG64
ARSI	arylsulfatase I	Q5FYB1	SPG66
PGAP1	GPI inositol-deacylase	Q75T13	SPG67
KLC2	kinesin light chain 2	Q9H0B6	SPG68
RAB3GAP2	rab3 GTPase-activating protein non-catalytic subunit	Q9H2M9	SPG69
MARS	methionine--tRNA ligase, cytoplasmic	P56192	SPG70
ZFR	zinc finger RNA-binding protein	Q96KR1	SPG71
REEP2	receptor expression-enhancing protein 2	Q9BRK0	SPG72
CPT1C	carnitine O-palmitoyltransferase 1, brain isoform	Q8TCG5	SPG73
IBA57	putative transferase CAF17, mitochondrial	Q5T440	SPG74
MAG	myelin-associated glycoprotein	P20916	SPG75
CAPN1	calpain-1 catalytic subunit	P07384	SPG76
FARS2	phenylalanine--tRNA ligase, mitochondrial	O95363	SPG77
ATP13A2	cation-transporting ATPase 13A2	Q9NQ11	SPG78
UCHL1	ubiquitin carboxyl-terminal hydrolase isozyme L1	P09936	SPG79
UBAP1	ubiquitin-associated protein 1	Q9NZ09	SPG80
TPP1	tripeptidyl-peptidase 1	O14773	-

**Table S5. Overlap of protein composition within the TS cluster, related to Figure 4**		
	T	S
T		100.0%
S	82.1%	

**Table S6. Overlap of protein composition within the EPOD cluster, related to Figure 4**				
	E	P	D	O
E		60.3%	96.5%	81.0%
P	38.6%		36.8%	92.9%
D	48.2%	28.8%		45.2%
O	29.8%	53.4%	33.3%	

**Table S7. Overlap of protein composition between the TS and the EPOD cluster, related to Figure 4**						
	E	P	T	S	D	O
E			96.4%	100.0%		
P			17.9%	21.7%		
T	23.7%	6.8%			43.9%	4.8%
S	20.2%	6.8%			40.4%	4.8%
D			89.3%	100.0%		
O			7.1%	8.7%		
