# Supplementary material for: Integrating protein networks and machine learning for disease stratification in the Hereditary Spastic Paraplegias
Source: iScience. 2021 Apr 28;24(5):102484. doi: 10.1016/j.isci.2021.102484 (PMC8169945; doi:10.1016/j.isci.2021.102484)
Supplement: Document S2. Article plus supplemental information [file mmc7.pdf]

iScience

CellPress
OPEN ACCESS

Article

Integrating protein networks and machine learning for disease stratification in the Hereditary Spastic Paraplegias

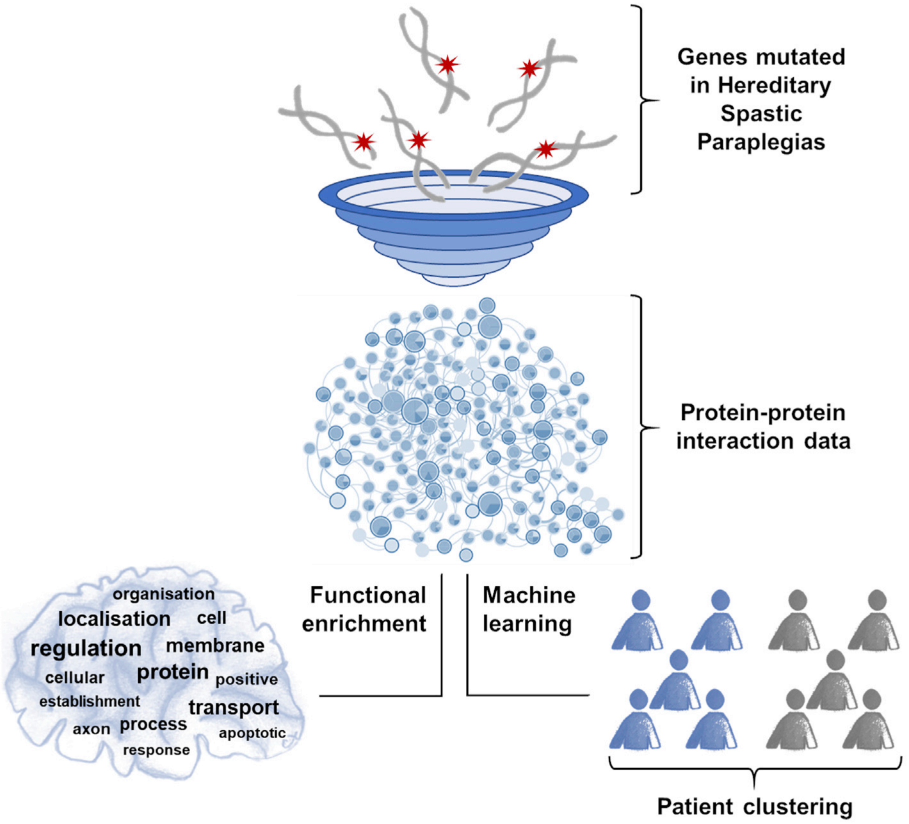

Nikoleta Vavouraki, James E. Tomkins, Eleanna Kara, ..., Marcus J. Tindall, Patrick A. Lewis, Claudia Manzoni

c.manzoni@ucl.ac.uk

Highlights

A bioinformatic study of the Hereditary Spastic Paraplegias using protein networks.

Human and manually curated protein-protein interaction data acquired using PINOT.

Intracellular transport and vesicle trafficking are suggested as disease mechanisms.

Machine learning techniques propose a patient clustering.

Vavouraki et al., iScience *24*, 102484
May 21, 2021 © 2021 The Authors.
https://doi.org/10.1016/j.isci.2021.102484

Check for updates

iScience

CellPress
OPEN ACCESS

Article

Integrating protein networks and machine learning for disease stratification in the Hereditary Spastic Paraplegias

Nikoleta Vavouraki,[1,2] James E. Tomkins,[1] Eleanna Kara,[3] Henry Houlden,[4] John Hardy,[3,5,6,7,8,9] Marcus J. Tindall,[2,10] Patrick A. Lewis,[1,3,9,11] and Claudia Manzoni[1,12,13,*]

SUMMARY

The Hereditary Spastic Paraplegias are a group of neurodegenerative diseases characterized by spasticity and weakness in the lower body. Owing to the combination of genetic diversity and variable clinical presentation, the Hereditary Spastic Paraplegias are a strong candidate for protein-protein interaction network analysis as a tool to understand disease mechanism(s) and to aid functional stratification of phenotypes. In this study, experimentally validated human data were used to create a protein-protein interaction network based on the causative genes. Network evaluation as a combination of topological analysis and functional annotation led to the identification of core proteins in putative shared biological processes, such as intracellular transport and vesicle trafficking. The application of machine learning techniques suggested a functional dichotomy linked with distinct sets of clinical presentations, indicating that there is scope to further classify conditions currently described under the same umbrella-term of Hereditary Spastic Paraplegias based on specific molecular mechanisms of disease.

INTRODUCTION

The Hereditary Spastic Paraplegias (HSPs) are a group of heterogeneous neurodegenerative diseases characterized by the core features of slowly progressive bilateral lower limb spasticity, hyperreflexia, and extensor plantar responses (Harding, 1983) accompanied by degeneration of the upper motor neurons (Deluca et al., 2004). Although the first description of clinical presentations we now refer to as HSPs dates back at least 140 years (Strümpell, 1880; Lorrain, 1898), the molecular mechanisms responsible for disease onset are, to date, still unclear. A number of mechanisms have been proposed to contribute to the degenerative process, including dysfunction of intracellular active transport and endolysosomal trafficking, alteration of lipid metabolism and endoplasmic reticulum (ER) shaping, as well as disruption of mitochondria homeostasis (Blackstone, 2012, 2018a; Blackstone et al., 2011; Boutry et al., 2019).

The heterogeneity of the HSPs derives from both the complex range of clinical presentations (summarized in Table S1) and diverse underlying genetic causes. Regarding the former, the age of onset can vary from early childhood to late adulthood, all modes of inheritance can be observed, and the form of the disease can be pure or complex. Complex forms of the HSPs are defined by the co-occurrence of clinical features in addition to lower limb spasticity, including peripheral neuropathy, seizures, cognitive impairment, and optic atrophy (Fink, 2013). Regarding the genetic heterogeneity of HSPs, mutations in over 70 genes have been associated with the HSPs (Faber et al., 2017), rendering it one of the hereditary disorders with the highest numbers of known causative genes (Blackstone, 2018a). In such a complex scenario, it is not clear as to whether all the HSP syndromes, despite being classified under the same umbrella term, share the same underlying molecular etiology (Blackstone, 2018a). Given the lack of treatments able to prevent, halt, or revert the HSPs, understanding the etiology of these disorders and gaining greater clarity in this area of HSP biology is crucial.

The intersection of genetics and functional biology has, historically, been dominated by single gene investigations, focusing on understanding the role of individual genes in cellular processes and phenotypes. This approach is powerful, but it allows for studying a limited number of genes at a time (Manzoni et al., 2020). In contrast, systems biology approaches such as protein-protein interaction (PPI) network (PPIN)

[1]School of Pharmacy, University of Reading, Reading, RG6 6AX, UK

[2]Department of Mathematics and Statistics, University of Reading, Reading, RG6 6AX, UK

[3]Department of Neurodegenerative Disease, UCL Queen Square Institute of Neurology, London, WC1N 3BG, UK

[4]Department of Neuromuscular Diseases, UCL Queen Square Institute of Neurology, London, WC1N 3BG, UK

[5]UK Dementia Research Institute at UCL and Department of Neurodegenerative Disease, UCL IoN, UCL London, W1T 7NF UK

[6]Reta Lila Weston Institute, UCL IoN, 1 Wakefield Street, London, WC1N 1PJ, UK

[7]UCL Movement Disorders Centre, Department of Clinical and Movement Neurosciences, UCL Queen Square Institute of Neurology, London, WC1N 3BG, UK

[8]Institute for Advanced Study, The Hong Kong University of Science and Technology, Hong Kong SAR, China

[9]Aligning Science Across Parkinson's (ASAP) Collaborative Research Network, Chevy Chase, MD 20815, USA

[10]Institute of Cardiovascular and Metabolic Research, University of Reading, Reading, RG6 6AS, UK

[11]Department of Comparative Biomedical Sciences, Royal Veterinary College, London, NW1 0TU, UK

[12]School of Pharmacy, University College London, London, WC1N 1AX, UK

[13]Lead contact

Continued

 © 2021 The Authors.
This is an open access article under the CC BY license (http://creativecommons.org/licenses/by/4.0/).

analyses provide tools to evaluate the entirety of known genes/proteins involved in a disease collectively through a holistic approach (Koh et al., 2012). The connections within the PPIN can be subjected to mathematical analysis to gain insight into the global relationships among potential contributors to the disease process, thus creating an *in silico* model system to investigate the molecular mechanisms and generate hypotheses to further support functional research and disease modeling (Manzoni et al., 2020).

This article describes the first study in which PPINs are created solely based on experimentally validated human PPIs of HSP genes and are applied to the investigation of HSP pathogenesis to identify global mechanisms, as well as individual processes involved in subtypes of disease following stratification based on the association of specific HSP genes with particular clinical features. Based on a combination of network, functional, and machine learning analyses, we propose HSPs to be subdivided into at least two major etiological groups. These results might suggest that not all the HSPs' clinical manifestations relate to the same disruption at a molecular level, and that it is indeed possible to hypothesize stratification of patients with HSP based on the molecular aspects of disease. This is an *in silico* modeling approach, thus it would require further functional validation; nevertheless, it suggests that both drug discovery and clinical trials for HSPs would need to take into consideration the molecular heterogeneity of disease.

RESULTS

Generation of PPI networks

The HSP seeds (HSP genes, n = 66 and test seeds, n = 17; see Table S2 and STAR Methods for more details) were used as the input list to query the online tool, PINOT (Tomkins et al., 2020), generating a list of experimentally validated human PPIs. Briefly, PINOT collects PPIs from seven manually curated databases that fully or partially comply with the IMEx annotation guidelines (Orchard et al., 2012) and scores each interaction based on the number of different methods and publications in which it has been described. PPIs with a final PINOT score <3 were excluded from further analyses as these interactions lack replication in the curated literature (i.e., they are reported in only one publication and detected by only one method). Following this filter, 746 interactors of HSP seeds were retained. Of note, 15 of the initial seeds were excluded due to no PPIs being identified (a total of 57 HSP seeds and 11 test seeds were retained). The resulting filtered network was termed the global HSP-PPIN and was composed of 814 nodes (57 HSP seeds + 11 test seeds + 746 interactors) connected via 925 edges (Data S1). The global HSP-PPIN (Figure S1) was composed of 1 main graph that contained the majority of nodes (n = 755/814, 92.8%), including the majority of seeds (n = 53/68, 77.9%) and 14 additional unconnected, smaller graphs. Of particular note is the presence of an interactor in the global HSP-PPIN, RNF170, which was found to be associated with the HSPs (i.e., an additional HSP gene) in a study published after the creation of the network (Wagner et al., 2019).

Each protein of the global HSP-PPIN was scored based on the number of seeds to which it was directly connected, and a degree distribution was plotted (Figure S2). All nodes interacting with at least 2 seeds (IIHs) were selected and used to extract the core HSP-PPIN composed of 164 nodes (including 45/57 HSP seeds [72.7%] and 8/11 test seeds [78.9%]) and 287 edges (Figure 1 and Data S2). The core HSP-PPIN represents the most interconnected part of the global HSP-PPIN graph and contains the interactors that are communal to 2 or more seeds, thus it can be used to investigate common functionalities across the different HSP genes.

Of note, the test seed CCDC50 is present in the core HSP-PPIN and directly interacts with 2 proteins that are interactors of 6 HSP seeds. Comparatively, 95.5% of the proteins within the global HSP-PPIN and 74.5% of the proteins within the core HSP-PPIN interacted with less than 6 HSP seeds. The strong connectivity of CCDC50 with HSP seeds indicates that they might be functionally related, and thus further supports the hypothesis that CCDC50 could be an HSP gene based on its genetic location (CCDC50 is located at 3q28 [NCBI gene: https://www.ncbi.nlm.nih.gov/gene/152137], whereas the genetic locus of SPG14 is 3q27-28 [Boutry et al., 2019]).

Functional enrichment: trafficking and intracellular organization

The nodes composing the core HSP-PPIN were analyzed through functional enrichment to identify associated Gene Ontology Biological Processes (GO-BPs). Three different enrichment tools were used (g:Profiler, PantherGO, and WebGestalt; Table S3). Despite p values being corrected differently in the different tools, the enrichment ratio was calculated using the same formula (see STAR Methods). We therefore selected the top 10 GO-BP terms (based on the enrichment ratio) from each of the 3 tools (Figure 2).

*Correspondence:
c.manzoni@ucl.ac.uk
https://doi.org/10.1016/j.isci.2021.102484

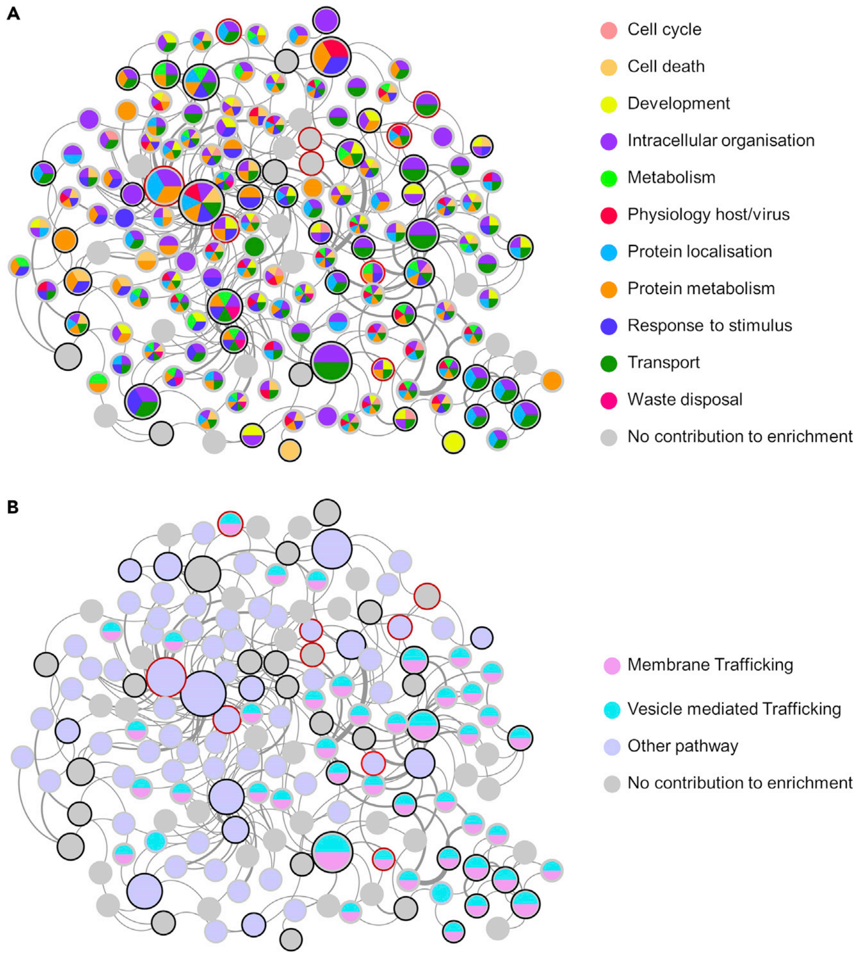

Figure 1. Functional enrichment of the core HSP-PPIN

The core HSP-PPIN is the most interconnected part of the global HSP-PPIN and includes (1) the interactors connecting at least 2 seeds and (2) the connected seeds. Seeds (HSP genes) are represented with a black border and test seeds with a red border (ACO2, ALS2, BICD2, CCDC50, CCT5, IFIH1, KIDINS220, LYST). The size of each node positively correlates with its number of connections (i.e., node degree) within the core HSP-PPIN. The thickness of each edge positively correlates with the final score of the respective interaction as calculated by PINOT (which is a proxy for confidence as it represents the sum of the number of different publications and number of different methods reporting the interaction).

(A) Nodes contributing to the enrichment of functional blocks (built on Gene Ontology Biological Processes) are color coded according to the legend (gray nodes are those that did not contribute to any of the enriched functional blocks).

(B) The involvement of nodes of the core HSP-PPIN in pathways is visualized by node colour coding based on Reactome's pathway analysis. (See also Figures S1 and S2 and Table S2).

The majority of the top terms indicated functions such as those of "Transport" or "Intracellular organisation" (collectively accounting for 60%–70% of terms significantly enriched using the 3 tools). The remaining terms referred to "Cell death" and "Physiology-host/virus" with important reference to protein targeting and the endomembrane system. Of note, we observed a 60% match of the GO-BPs in the top 10 enriched terms across all the 3 tools and 60%–100% match between at least 2 tools (g:Profiler: 100%, WebGestalt: 100%, and PantherGO: 60%). The unique terms from each tool, however, were closely related to already shared terms (e.g., "Anterograde axonal transport" [unique to PantherGO] is closely related to "Retrograde neuronal dense core vesicle transport" and "Retrograde axonal transport" [g:Profiler, WebGestalt and PantherGO]) (Figure 2).

The entirety of enriched GO-BP terms was then grouped by semantic similarity into semantic classes, which were further organized into functional blocks, thus aiding the interpretation of the enrichment results (see STAR Methods and Bonham et al., 2018; Ferrari et al., 2018; Ferrari et al., 2017; Tomkins et al., 2018).

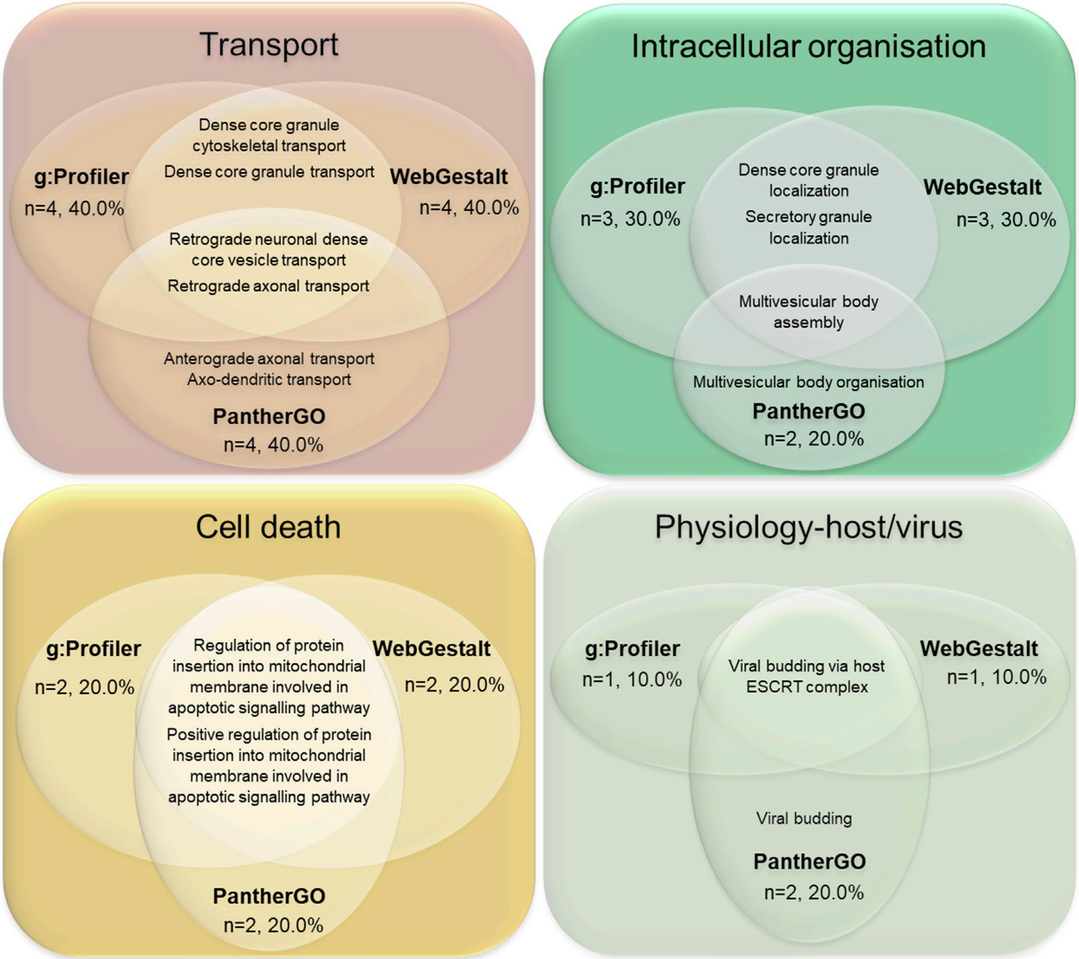

Figure 2. Top 10 GO-BPs enriched within the core HSP-PPIN

The 10 GO-BP terms from the functional enrichment of the core HSP-PPIN with the highest enrichment ratio were grouped into functional blocks based on semantic similarity. Most of the terms resulted from at least 2 enrichment tools (g:Profiler & WebGestalt: n = 10/10, 100%; PantherGO: n = 6/10, 60%).

The raw results from each tool were similar in all three levels explored: the identity of the GO-BP terms, of the semantic classes, and of the functional blocks. In fact, most of the GO-BP terms were common to at least 2 tools (n = 115/171, 67.3%) (Figure S3A), whereas after semantic classification of GO-BPs a higher proportion of semantic classes derived from at least 2 tools (n = 49/58, 84.5%) (Figure S3B). Finally, all the functional blocks were represented by all the 3 tools (n = 11/11, 100.0%) (Figure S3C). Overall, this confirmed the consistency of results across different enrichment tools. However, these results also showed that even if consistency is very high at the more general levels of semantic classes and functional blocks, discrepancies can occur at the very specific GO-BP term level. Therefore, we decided to improve functional interpretation and reduce tool-specific bias in further analyses by merging the GO-BP terms derived from the 3 tools within functional blocks replicated in at least 2 tools (in this case all terms) and adjusting the threshold of the p value (see STAR Methods).

The majority of significant GO-BP terms from the core HSP-PPIN enrichment analysis were associated with the functional block "Intracellular organisation" (22.2%), followed by "Transport" (19.3%), and then "Protein localisation" (13.5%), collectively accounting for more than half of GO terms (55.0%) (Figures 3 and S4, Table S3). This result confirmed the findings previously obtained from the top 10 enriched terms, suggesting a role for these processes in the molecular mechanism(s) underlying HSP pathogenesis. Finally, and to overcome any bias based on the architecture of the grouping of GO terms, we also performed text mining for single key words within all the significantly enriched GO-BP terms and detected significant enrichment for "axon" (n = 7/171, 4.1% [8.9-fold enrichment] $p < 10^{-10}$ after 1,000 random simulation),

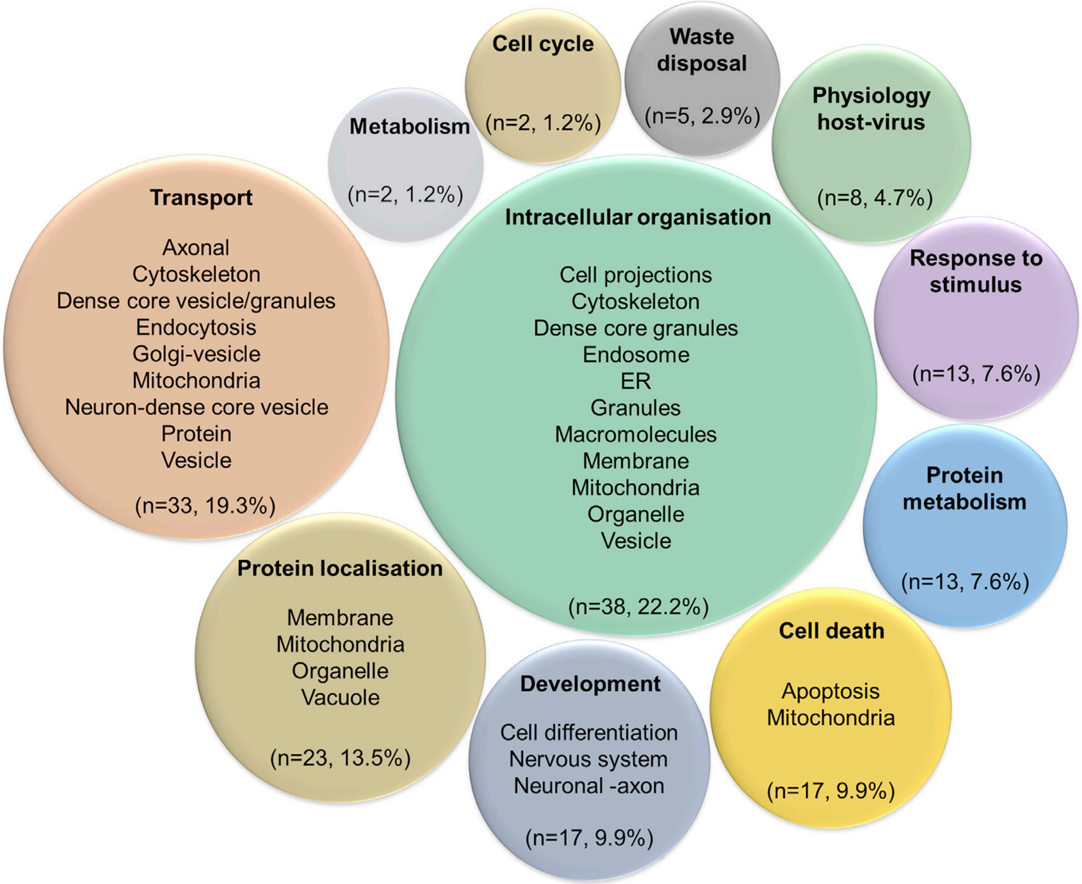

Figure 3. Graphical representation of the functional enrichment of the core HSP-PPIN
Functional enrichment was performed on the nodes of the core HSP-PPIN. The resulting GO-BP terms (n = 171) (Table S3) were grouped into semantic classes (brief descriptions of several semantic classes are inside each circle) and then into functional blocks (title of each circle, bolded). The number and percentage of terms in each functional block was calculated for g:Profiler, WebGestalt, and PantherGO as described in STAR Methods. For a more detailed version see Figure S4.

"endosomes" (n = 3/171, 1.8% [5.7-fold enrichment] $p < 10^{-10}$), "membrane" (n = 24/171, 14.0% [5.7-fold enrichment], $p < 10^{-10}$), "neurons" (n = 9/171, 5.3% [3.4-fold enrichment], p = 7.85 10^{-7}), "projection" (n = 6/171, 3.5% [5.4-fold enrichment], p = 6.54 10^{-7}), and "vesicles" (n = 10/171, 5.8% [4.5-fold enrichment], $p < 10^{-10}$).

Of note, the independent analysis of the core HSP-PPIN through Reactome (Table S3) suggested similar enrichment, whereby the 2 most significantly enriched pathways were vesicle-mediated transport (REA identifier: R-HSA-5653656, $p < 10^{-10}$, 46 (28.0%) contributing nodes) and membrane trafficking (REA identifier: R-HSA-199991, $p < 10^{-10}$, 44 (29.3%) contributing nodes) (Figure 1B).

Stratification of HSP clinical groups into two clusters

HSPs can present with a wide set of clinical features, with marked phenotypic heterogeneity between different patients. The complex forms of HSPs are defined by the co-occurrence of additional clinical features, the most frequently reported being peripheral neuropathy (P), thinning of the corpus callosum (T), seizures (S), dementia or mental retardation (D), and optic atrophy (O). Finally, some patients also present with an early disease onset (E). Interestingly, medical reports and case studies sometimes state the presence of the aforementioned features in association with specific mutations in HSP genes. We have taken advantage of this knowledge and grouped the genes based on the features with which they are associated. Therefore, the seeds within the core HSP-PPIN were coded based on their associated clinical features (Figure S5). Of note, some seeds are associated with a single feature (n = 9/57, 16%), whereas others are

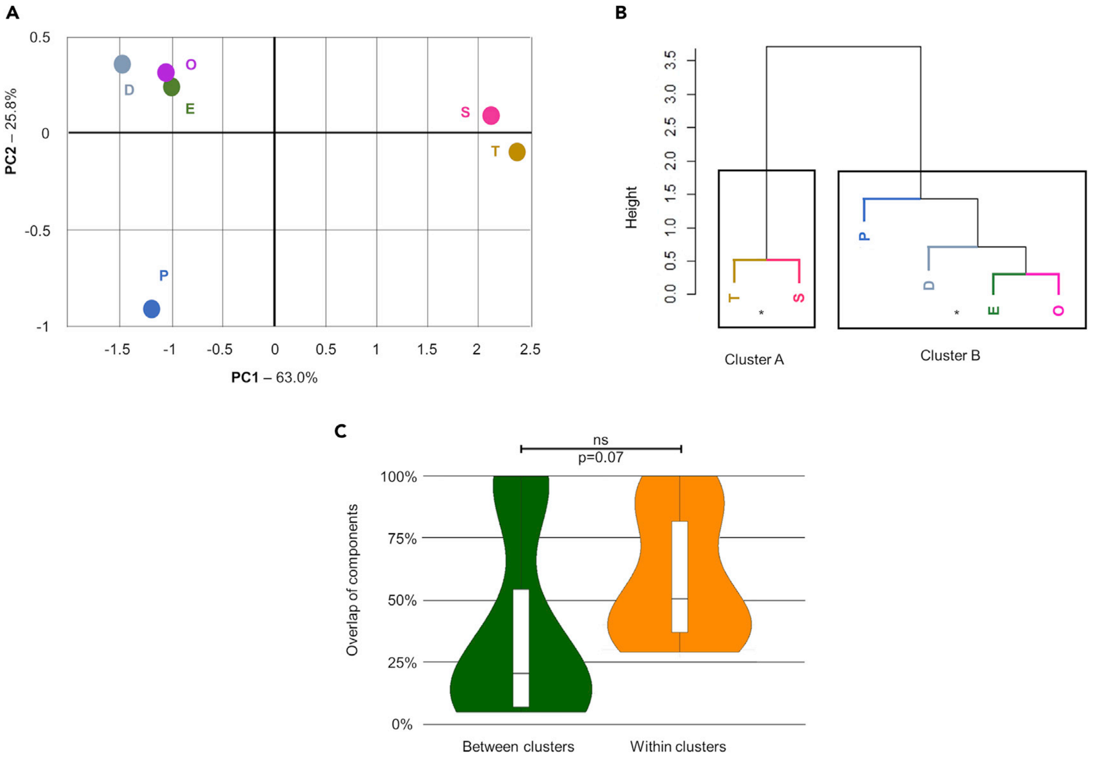

Figure 4. Comparison of the functional profiles of the six clinical subnetworks

(A) In the PCA graph each clinical subnetwork is represented by a single point of coordinates calculated based on PCA performed for the percentage of GO-BP terms and adjusted based on the explained variation of each axis (for details see STAR Methods) [i.e., (x, y) = (PC1x0.630, PC2x0.258)].

(B) Cluster dendrogram produced based on hierarchical clustering of the gene groups as analyzed in (A), in which the two suggested clusters are shown. *pvclust-p-value>0.90 (pvclust-p-value A = 0.99, pvclust-p-value B = 0.91). E, early onset; P, peripheral neuropathy; T, thin corpus callosum; S, seizures; D, dementia or mental retardation; O, optic atrophy.

(C) The percentage of protein identity between gene groups within the same cluster (EPOD and TS cluster) was compared with the protein identity between gene groups of different clusters using t-test (two-tailed, unequal distribution). (See also Figures S7–S10 and Tables S5–S7).

responsible for 2 (n = 18/57, 32%), 3 (n = 12/57, 21%), or 4 (n = 7/57, 12%) clinical features. This seed characterization allowed the extraction of 6 smaller subnetworks from the core HSP-PPIN, each of them containing the interconnected seeds (and their interactors) associated with each specific feature mentioned above (Figure S6).

Enrichment of biological processes was performed on each clinical subnetwork separately, as previously described, using g:Profiler, PantherGO, and WebGestalt (Table S4 and Figure S7). The enrichment results obtained from the 3 tools were compared to assess their reproducibility and identify GO-BP terms of functional blocks that were replicated in at least 2 tools. These terms were merged to increase functional coverage as described earlier. The percentage of GO-BP terms within each functional block was calculated to weight its relevance. Principal-component analysis (PCA) was then applied to reduce the complexity of the results obtained from the functional enrichment analyses to 2 principal components (PC1 and PC2). PCA thus allowed comparison of the 6 clinical subnetworks (Figure 4A). Interestingly, some of the clinical subnetworks functionally clustered together. Of note, this result was obtained with PCA performed on both the percentage of the GO terms in each functional block (Figure 4A) and their absolute numbers (Figure S8A).

The PCA plot provided a first visual insight into potential functional clustering that was further confirmed by hierarchical clustering. Results were plotted into a cluster dendrogram (Figures 4B and S9), and the exact number of clusters to best fit the data was determined by 2 methods: Silhouette method and Multiscale bootstrap resampling (Figure S10). Both methods suggested the presence of 2 clusters (named clusters A and B) in the cluster dendrogram (Silhouette method: the highest score was for 2 clusters; Multiscale

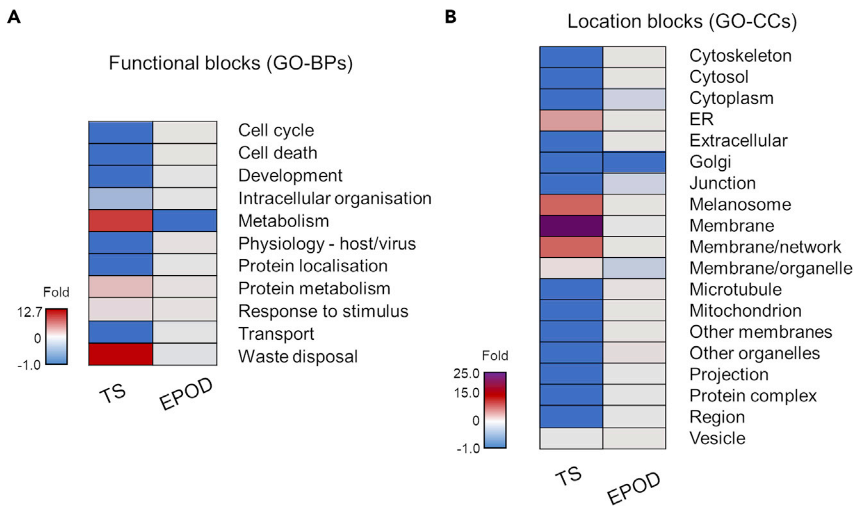

Figure 5. Differential patterns of enrichment for the TS and EPOD clusters

(A and B) The distribution of the GO-BP terms (A) and GO-CC terms (B) of the clusters, TS and EPOD, are presented as a fold change compared with the profile of the core HSP-PPIN. A more detailed version is shown in Figure S11, whereas the totality of the results is shown in Table S8.

bootstrap resampling: Clusters A and B had a pvclust-p-value = 0.99 and 0.91, respectively, showing 99% and 91% confidence in the result). Cluster A is composed of thin corpus callosum and seizures (hereafter named TS), whereas cluster B is composed of early onset, peripheral neuropathy, optic atrophy, and dementia or mental retardation (hereafter named EPOD).

The co-clustering of the T and S subnetworks within the TS cluster is not surprising as they had 23 common proteins (n = 23; T∩S = 82.1%, S∩T = 100%). However, we also observed a large overlap of proteins between the subnetworks of O and P (n = 39; O∩P = 92.9%, P∩O = 53.4%), T and D (n = 25; T∩D = 89.3%, D∩T = 43.9%), S and E (n = 23; S∩E = 100%, E∩S = 20.2%), and D and E (n = 55; D∩E = 96.5%, E∩D = 48.2%). In all these cases, the common composition was large, yet not able to guide the order of similarity based on the dendrogram, or to promote the co-clustering (Figure 4B). A full report of the overlaps between the clinical subnetworks is detailed in Tables S5–S7.

Plotting the percentages of overlaps across different clinical subnetworks allowed for running a statistical comparison. When considering the overlap of the subnetworks within cluster TS and within cluster EPOD (networks within the same cluster) in comparison to the overlaps of the subnetworks in TS versus EPOD (networks in different clusters) we found a non-significant difference in their distributions ($p = 0.07$; Figure 4C). This result suggests that the generation of the 2 distinct clinical clusters was highly affected by similarities in the functional profile of the subnetworks in terms of GO-BPs, whereas the overlap of nodes had a small or potentially no contribution.

Differences between the clinical clusters based on functions and subcellular localization

The potential differences of the 2 clinical clusters were further explored by performing enrichment analysis for GO-BPs using as input the protein components of the 2 clusters, TS and EPOD (Table S8). The comparison of the 2 obtained functional profiles is shown in Figures 5A and S11. Despite an overlap in the identity of the GO-BPs functional blocks between the 2 clusters (TS: n = 4/5, 80%; EPOD: n = 4/10, 40%), the granular distribution of specific GO-BP terms in each functional block differs between clusters, with the GO-BP functional blocks of: "Waste disposal" (+12.7-fold [compared with the core HSP-PPIN]), "Metabolism" (+9.3-fold), and "Protein metabolism" (+2.15-fold) being more represented in the TS rather than in the EPOD cluster (−0.13-, −1.0-, and 0.25-fold, respectively) (Figure 5A). Meanwhile, the GO-BP functional blocks "Physiology-host/virus" (+0.22-fold), "Cell cycle" (+0.1-fold), and "Cell death" (+0.1-fold) were more represented in the EPOD rather than in the TS cluster (−1.0-, −1.0-, and −1.0-fold, respectively). Interestingly, 5 GO-BP terms related to the unfolded protein response (e.g., "Cellular response to unfolded protein" and "Cellular response to topologically incorrect protein") were unique to the TS cluster (n = 5/25,

25%), even with cluster EPOD having a 6-fold higher number of total GO-BP terms ($n_{GO\text{-}BPtotalEPOD}$ = 158 versus $N_{GO\text{-}BPtotalTS}$ = 25), thus highlighting the importance of protein folding for the TS cluster only. Overall, these results of GO-BP enrichment indicated that functions associated with protein metabolism, waste disposal, and unfolded protein response might be more important processes in the TS rather than in the EPOD cluster, whereas the EPOD cluster presents with a functional enrichment profile very similar to that of the entire core HSP-PPIN.

Similarly, we performed Gene Ontology Cellular Component (GO-CC) enrichment using as input the protein components of the 2 clusters TS and EPOD (Table S8). The comparison of the 2 obtained cellular components profiles is shown in Figures 5B and S11, where location block is a sister term to the functional block of GO-BP terms. Even though there are common GO-CC location blocks between the 2 clusters (TS: n = 5/6, 83.3%; EPOD: n = 5/17, 29.4%), the composition of the most enriched location blocks based on the percentage of GO-CC terms differed substantially. Interestingly, and confirming the results obtained previously with GO-BPs, a higher percentage of GO-CC location blocks are related to "ER" (+4.7-fold [compared with the core HSP-PPIN]), "Melanosomes" (+8.5-fold), and "Membranes" (i.e., "Membranes": +25.0-fold, "Membrane/network" +8.5-fold, and "Membranes/organelle" +0.5-fold) for the TS cluster when compared with the EPOD cluster (0.13-, 0.13-, 0-, 0.13-, −0.30-fold, respectively). As for the EPOD clusters, higher enrichment is observed in the GO-CC location blocks: "Other organelles" (+0.5-fold), "Microtubules" (+0.4-fold), "Cytoskeleton," "Cytosol," "Extracellular," "Mitochondria," and "Other membranes" (+0.1-fold) than in TS (-1-fold for all these location blocks in TS).

DISCUSSION

Network-based approaches have been increasingly used to study complex human diseases, such as neurodegenerative diseases and cancer (Manzoni et al., 2020). The HSPs are neurodegenerative diseases with considerable genetic and clinical heterogeneity (Boutry et al., 2019; Faber et al., 2017), rendering them particularly interesting to study using a PPIN approach. We applied a bottom-up approach, starting with the selection of genes involved in the disease and built the relevant interactome around them. We focused on experimentally validated human PPIs of HSP genes, not including genes associated with a disease spectrum in which HSP is involved (e.g., HSP-ataxia spectrum) or genes with related phenotype, in contrast with prior studies (Parodi et al., 2018; Novarino et al., 2014; Synofzik and Schule, 2017; Bis-Brewer et al., 2019). Although the excluded data might be useful in the effort to conceptualize the possible interactions and mechanisms of HSP-related diseases, they were not considered to be specific or supported strongly enough to be included in our analysis.

We applied the PINOT pipeline to mine the curated literature and download PPIs for each single seed, thus obtaining each seed's interactome (Ferrari et al., 2018). We then constructed the global HSP-PPIN by combining each seed's interactome in a modular fashion. We finally filtered the global HSP-PPIN, excluding the nodes that interacted with a single seed, thus retaining those interactors that were bridging at least 2 seeds' interactomes. This step allowed for removal of all the unique interactors of each seed and for the extraction of the core HSP-PPIN, which is the most connected part of the network, containing nodes that are shared across seeds, and responsible for connections across different interactomes. By containing all the shared interactors and connections among seeds, the core HSP-PPIN can be used to infer shared functions communal to multiple HSP genes (Tomkins et al., 2020).

It is important to observe that most HSP seeds are indeed part of the core HSP-PPIN, meaning they are connected through at least one shared interactor. This result suggests that they are likely to be functionally related (based on the guilt-by-association principle [Oliver, 2000]) and therefore convergent molecular mechanism(s) drive disease pathogenesis, regardless of the mutated gene acting to initiate the degenerative process. The seeds that were absent from the core HSP-PPIN (i.e., seeds that do not share any interactors with other seeds) had a low number of curated interactors ranging from 0 to 4 (*PLA2G6, CPT1C, CYP2U1, C12orf65, B4GALNT1, TECPR2, ENTPD1, ATL1, SPG11, DDHD1, AP5Z1, SLC16A2, GAD1, RAB3-GAP2*, and *HACE1*). With limited interactors, their absence from the core HSP-PPIN could be the result of ascertainment bias (i.e. these seeds are understudied proteins with limited number of known interactors) rather than representing a more fundamental divergence in etiology (Schaefer et al., 2015). As more PPIs are discovered, the human interactome will become more complete (Luck et al., 2020; Rolland et al., 2014; Huttlin et al., 2017; Wewer Albrechtsen et al., 2018) and might be able to help us better understand the connecting processes of large groups of genes and potentially point toward the disease

mechanism. Exceptions were EXOSC3 (test seed), SPG21 (HSP-seed), and KCNA2 (test seed) with 21, 10, and 6 interactors, respectively. In this second scenario, it can be hypothesized that these seeds are not interacting with other HSP seeds, meaning that, by not sharing the same interactome, they might potentially be associated with different molecular mechanisms of disease.

In this study we included 17 test seeds, genes for which there is no clear consensus regarding their potential association with HSPs, as they have been controversially reported in clinical literature. Eight of the test seeds (i.e., ALS2, BICD2, CCDC50, CCT5, KIDINS220, ACO2, LYST, and IFIH1) were present in the core HSP-PPIN, providing *in silico* evidence of their relevance within the HSP protein interaction landscape. The presence of five of those test seeds (i.e., CCT5, KIDINS220, ACO2, LYST, and IFIH1) correlates with the processes and cellular components indicated to play a role in HSPs from previous and the current work, namely, of lysosomal homeostasis, protein folding and transport, cell death, neurodegeneration, and antiviral responses, with which they also have been associated (Crow et al., 2020; Faigle et al., 1998; Freund et al., 2014; Leong and Chow, 2006; Liao et al., 2007; Spiegel et al., 2012). The presence of ALS2 in the core HSP-PPIN is not surprising, as it is considered an HSP gene by many clinicians and researchers (Lo Giudice et al., 2014; Boutry et al., 2019; de Souza et al., 2017). An interesting test seed present in the core HSP-PPIN is CCDC50, because it was included in this study based on its chromosomal location being within the locus of SPG14 (CCDC50 is located at 3q28 [NCBI gene: https://www.ncbi.nlm.nih.gov/gene/152137], whereas the genetic locus of SPG14 is 3q27-28 [Boutry et al., 2019]). Of note, CCDC50 formed interactions with more seeds than most interactors of the global HSP-PPIN and the core HSP-PPIN. This result represents an *in silico* prediction that alterations in CCDC50 could be leading to the HSP type SPG14 and it suggests to include CCDC50 in the list of prioritized genes to be screened for rare variant discovery.

Notably, the protein product of the gene *RNF170* was found to be associated with HSPs (and published) after this analysis commenced (Wagner et al., 2019) and was indeed present within the global HSP-PPIN. This result demonstrates the utility in using PPINs to study complex disorders, as they can aid prioritization of candidate genes from genetic analysis (Erlich et al., 2011) and hint to key proteins involved in disease mechanisms.

The analysis of a disease-focused PPIN based on functional annotation provides an opportunity to gain a deeper understanding of the underlying mechanism(s) of disease using a holistic view (Koh et al., 2012). Therefore, enrichment analysis was performed for the components of the core HSP-PPIN, supporting the involvement of some of the processes previously suggested to be associated with the disease mechanism of HSPs. Of the 10 mechanisms suggested by Lo Giudice et al. (2014), 3 were supported by the results of this work, namely, "endosome membrane trafficking and vesicle formation," "abnormal membrane trafficking and organelle shaping," "dysfunction of axonal transport," and also 3 additional processes, namely, "autophagy," "axon development," and "abnormal cellular signaling in protein morphogenesis," whereas we did not find evidence in our analysis for "oxidative stress," "abnormal lipid metabolism," "abnormal DNA repair," and "dysregulation of myelination." Regarding the mechanisms hypothesized by de Souza et al. (2017), those in accordance with this work were "intracellular active transport," "endolysosomal trafficking pathways," and "ER shaping," whereas we did not find evidence in our analysis for "lipid metabolism," "mitochondrial dysfunction," or "migration and differentiation of neurons." Our results are more in line with the suggestion from Blackstone (2018a) that the key biological processes at play in the etiopathogenesis of HSPs are "organelle shaping and biogenesis" and "membrane cargo and trafficking," further supporting the notion that HSPs could be considered transportopathies (Gabrych et al., 2019), and that the dysregulation of ER morphology and function could be implicated in HSPs (Lee and Blackstone, 2020). However, some of the suggested hypotheses, namely, "nucleotide metabolism," "mitochondrial function," and "lipid/cholesterol metabolism" (Blackstone, 2018a), were not supported by the findings of this study. Interestingly, functional data were not used for the creation of the HSP-PPINs, therefore the conclusions obtained here are only based on PPIs and represent a further validation of some of the published functional analyses. These results highlight the potential of a PPIN analysis approach combined with functional enrichment to identify the most relevant functions among the genes of interest related to a complicated disease, which is an important step for discovering disease-modifying agents. A similar approach has been used in Ferrari et al. (2018) to compare the functional profiles of Mendelian Parkinson disease, parkinsonism, and frontotemporal dementia genes. In Dervishi et al. (2018) PPIN analysis coupled with expression profiling was used to isolate key cellular events in amyotrophic lateral sclerosis, whereas Bonham et al. (2018, 2019) applied protein networks for the functional evaluation of behavioral and language variant frontotemporal dementia.

To explore if the clinical diversity of the HSPs reflects a mechanistic heterogeneity of disease, machine learning tools (PCA and hierarchical clustering) were used to analyze the functional profile of the core HSP-PPIN. Based on our *in silico* analysis, we suggest the existence of at least 2 main subtypes of HSPs. The first functional subtype includes the clinical features of thin corpus callosum and seizures (i.e., TS cluster), whereas the second gathers those cases characterized by early onset, peripheral neuropathy, dementia or mental retardation, and optic atrophy (i.e., EPOD cluster). Further analysis for biological processes of the 2 clinical clusters suggested that "protein metabolism" and "waste disposal" are prominent in the TS cluster. In addition, most of the unique results for this cluster were related to the unfolded protein response. These results support the relevance of the regulation of protein level and conformation for the TS cluster. In contrast, for the EPOD cluster, the most important functions were related to "physiology-host/virus" and "cell death," which suggests that the endomembrane system involved in the viral process, together with mechanisms involved in cell survival, are of greater importance in the EPOD cluster.

These findings were further supported by cellular component and pathways analyses, where the TS cluster showed a higher enrichment in different types of membranes, melanosomes, and the ER, whereas results for the EPOD cluster were more focused on extracellular components, mitochondria, other organelles, and the cytoskeleton.

Therefore, this study provides a platform indicating that patients with HSP could be stratified based on the molecular mechanisms involved in disease etiopathogenesis, and this in turn can be beneficial for developing therapeutic strategies and aiding efforts to stratify patients for clinical trials.

This application provides insight into the utility of PPIN analysis in the study of complex disorders, as PPINs are a powerful tool that can extract and combine a large extent of previous data in a relatively quick and easy fashion. The use of this approach can create a comprehensive picture that summarizes the current knowledge, helping in prioritizing and confirming existing mechanistic theories, guiding research based on the identification of interesting proteins and pathways, as well as highlighting uncertain areas that require further investigation.

Limitations of the study

It is important to note the limitations of the approach used in this study. The mapping of the human interactome has progressed massively within the last decade, but it is still incomplete and for the most part it is still based on hypothesis-driven experiments. In addition, the most accurate and trustworthy type of curation for PPI data (i.e. manual curation) is also the most time-consuming, leading to a delay between the publishing of PPIs and their input in PPI databases. This introduces two typical biases of protein networks: they are incomplete by definition and affected by ascertainment bias. Another consideration that is worth raising is that some relevant pathways can be consequential to disease and therefore not directly dependent on the first layer of protein interactions built around the seeds.

As a result, PPI-based analyses are affected by type II error. In this specific case, for example, some functions genuinely associated with HSPs could be omitted from the results. It is also worth considering that the results presented in this study require further functional and clinical validation. At the same time, however, this study provides a platform indicating that patients with HSP could be stratified based on the molecular mechanisms involved in disease etiopathogenesis, and this in turn can be beneficial for developing therapeutic strategies and aiding efforts to stratify patients for clinical trials.

STAR★METHODS

Detailed methods are provided in the online version of this paper and include the following:

- KEY RESOURCES TABLE
- RESOURCE AVAILABILITY
  - Lead contact
  - Materials availability
  - Data and code availability
- METHOD DETAILS
  - Selection of seeds
  - Collection of PPIs and HSP-PPINs

- ○ Enrichment analyses
- ○ PCA & Hierarchical clustering
- ● QUANTIFICATION AND STATISTICAL ANALYSIS

SUPPLEMENTAL INFORMATION

Supplemental information can be found online at https://doi.org/10.1016/j.isci.2021.102484.

ACKNOWLEDGMENTS

N.V. is supported by the Engineering and Physical Sciences Research Council studentship (EP/M508123/1) and by the Dolby Family Fund.

J.E.T. is supported by the Biomarkers Across Neurodegenerative Diseases Grant Program 2019, BAND3 (Michael J. Fox Foundation, Alzheimer's Association, Alzheimer's Research UK, and the Weston Brain Institute [grant number 18063 awarded to C.M. and P.A.L.]) and previously by BBSRC CASE studentship BB/M017222/1 with BC Platforms.

E.K. is the recipient of an HFSP long-term fellowship (LT001044/2017).

This research was funded in whole or in part by Aligning Science Across Parkinson's [ASAP0478 to J.H. and P.A.L.] through the Michael J. Fox Foundation for Parkinson's Research (MJFF). For the purpose of open access, the author has applied a CC BY public copyright license to all Author Accepted Manuscripts arising from this submission. P.A.L. is supported by the MJFF.

Authors acknowledge additional support by the Medical Research Council (grant numbers MR/N026004/1 to J.H. and P.A.L.; MR/L010933/1 to P.A.L.); the Wellcome Trust to J.H. (grant number 202903/Z/16/Z); the National Institute for Health Research, University College London Hospitals Biomedical Research Centre to J.H.; the UK Dementia Research Institute (which receives its funding from Dementia Research Institute, funded by the UK Medical Research Council, Alzheimer's Society, and Alzheimer's Research UK); and the BRCNIHR Biomedical Research Center at University College London Hospitals NHS Foundation Trust and University College London.

AUTHOR CONTRIBUTIONS

Conceptualization, N.V., P.A.L., and C.M.; methodology, N.V. and C.M.; software, N.V., J.E.T., and C.M.; formal analysis, N.V.; resources, P.A.L. and C.M.; writing – original draft, N.V. and C.M.; writing – review & editing, N.V., J.E.T., E.K., H.H., J.H., M.J.T., P.A.L., and C.M.; visualization, N.V. and C.M.; supervision, E.K., H.H., J.H., M.J.T., P.A.L., and C.M.; funding acquisition, J.H., M.J.T., P.A.L., and C.M.

DECLARATION OF INTERESTS

The authors declare no competing interests.

Received: February 14, 2021
Revised: April 1, 2021
Accepted: April 23, 2021
Published: May 21, 2021

REFERENCES

Ahmed, M.Y., Al-Khayat, A., Al-Murshedi, F., Al-Futaisi, A., Chioza, B.A., Pedro Fernandez-Murray, J., Self, J.E., Salter, C.G., Harlalka, G.V., Rawlins, L.E., et al. (2017). A mutation of EPT1 (SELENOI) underlies a new disorder of Kennedy pathway phospholipid biosynthesis. Brain *140*, 547–554.

Akawi, N., Mcrae, J., Ansari, M., Balasubramanian, M., Blyth, M., Brady, A.F., Clayton, S., Cole, T., Deshpande, C., Fitzgerald, T.W., et al. (2015). Discovery of four recessive developmental disorders using probabilistic genotype and phenotype matching among 4,125 families. Nat. Genet. *47*, 1363–1369.

Ashburner, M., Ball, C.A., Blake, J.A., Botstein, D., Butler, H., Cherry, J.M., Davis, A.P., Dolinski, K., Dwight, S.S., Eppig, J.T., et al. (2000). Gene ontology: tool for the unification of biology. The Gene Ontology Consortium. Nat. Genet. *25*, 25–29.

Bis-Brewer, D.M., Danzi, M.C., Wuchty, S., and Zuchner, S. (2019). A network biology approach to unraveling inherited axonopathies. Sci. Rep. *9*, 1692.

Blackstone, C. (2012). Cellular pathways of hereditary spastic paraplegia. Annu. Rev. Neurosci. *35*, 25–47.

Blackstone, C. (2018a). Converging cellular themes for the hereditary spastic paraplegias. Curr. Opin. Neurobiol. *51*, 139–146.

Blackstone, C. (2018b). Hereditary spastic paraplegia. Handb Clin. Neurol. *148*, 633–652.

Blackstone, C., O'kane, C.J., and Reid, E. (2011). Hereditary spastic paraplegias: membrane traffic and the motor pathway. Nat. Rev. Neurosci. *12*, 31–42.

Bonham, L.W., Steele, N.Z.R., Karch, C.M., Broce, I., Geier, E.G., Wen, N.L., Momeni, P., Hardy, J., Miller, Z.A., Gorno-Tempini, M.L., et al. (2019). Genetic variation across RNA metabolism and cell death gene networks is implicated in the semantic variant of primary progressive aphasia. Sci. Rep. *9*, 10854.

Bonham, L.W., Steele, N.Z.R., Karch, C.M., Manzoni, C., Geier, E.G., Wen, N., Ofori-Kuragu, A., Momeni, P., Hardy, J., Miller, Z.A., et al. (2018). Protein network analysis reveals selectively vulnerable regions and biological processes in FTD. Neurol. Genet. *4*, e266.

Bouhouche, A., Benomar, A., Bouslam, N., Chkili, T., and Yahyaoui, M. (2006). Mutation in the epsilon subunit of the cytosolic chaperonin-containing t-complex peptide-1 (Cct5) gene causes autosomal recessive mutilating sensory neuropathy with spastic paraplegia. J. Med. Genet. *43*, 441–443.

Boutry, M., Morais, S., and Stevanin, G. (2019). Update on the genetics of spastic paraplegias. Curr. Neurol. Neurosci. Rep. *19*, 18.

Bouwkamp, C.G., Afawi, Z., Fattal-Valevski, A., Krabbendam, I.E., Rivetti, S., Masalha, R., Quadri, M., Breedveld, G.J., Mandel, H., Tailakh, M.A., et al. (2018). ACO2 homozygous missense mutation associated with complicated hereditary spastic paraplegia. Neurol. Genet. *4*, e223.

Breuer, K., Foroushani, A.K., Laird, M.R., Chen, C., Sribnaia, A., Lo, R., Winsor, G.L., Hancock, R.E., Brinkman, F.S., and Lynn, D.J. (2013). InnateDB: systems biology of innate immunity and beyond–recent updates and continuing curation. Nucleic Acids Res. *41*, D1228–D1233.

Clarencon, F., Touze, E., Leroy-Willig, A., Turmel, H., Naggara, O., Pavy, S., Brezin, A., and Mas, J.L. (2006). Spastic paraparesis as a manifestation of Leber's disease. J. Neurol. *253*, 525–526.

Crow, Y.J., Shetty, J., and Livingston, J.H. (2020). Treatments in aicardi-goutieres syndrome. Dev. Med. Child. Neurol. *62*, 42–47.

de Souza, P.V.S., De Rezende Pinto, W.B.V., De Rezende Batistella, G.N., Bortholin, T., and Oliveira, A.S.B. (2017). Hereditary spastic paraplegia: clinical and genetic hallmarks. Cerebellum *16*, 525–551.

Deluca, G.C., Ebers, G.C., and Esiri, M.M. (2004). The extent of axonal loss in the long tracts in hereditary spastic paraplegia. Neuropathol. Appl. Neurobiol. *30*, 576–584.

Dervishi, I., Gozutok, O., Murnan, K., Gautam, M., Heller, D., Bigio, E., and Ozdinler, P.H. (2018). Protein-protein interactions reveal key canonical pathways, upstream regulators, interactome domains, and novel targets in ALS. Sci. Rep. *8*, 14732.

Erlich, Y., Edvardson, S., Hodges, E., Zenvirt, S., Thekkat, P., Shaag, A., Dor, T., Hannon, G.J., and Elpeleg, O. (2011). Exome sequencing and disease-network analysis of a single family implicate a mutation in KIF1A in hereditary spastic paraparesis. Genome Res. *21*, 658–664.

Faber, I., Pereira, E.R., Martinez, A.R.M., Franca, M., Jr., and Teive, H.a.G. (2017). Hereditary spastic paraplegia from 1880 to 2017: an historical review. Arq. Neuropsiquiatr. *75*, 813–818.

Faigle, W., Raposo, G., Tenza, D., Pinet, V., Vogt, A.B., Kropshofer, H., Fischer, A., De Saint-Basile, G., and Amigorena, S. (1998). Deficient peptide loading and MHC class II endosomal sorting in a human genetic immunodeficiency disease: the Chediak-Higashi syndrome. J. Cell Biol. *141*, 1121–1134.

Ferrari, R., Kia, D.A., Tomkins, J.E., Hardy, J., Wood, N.W., Lovering, R.C., Lewis, P.A., and Manzoni, C. (2018). Stratification of candidate genes for Parkinson's disease using weighted protein-protein interaction network analysis. BMC Genomics *19*, 452.

Ferrari, R., Lovering, R.C., Hardy, J., Lewis, P.A., and Manzoni, C. (2017). Weighted protein interaction network analysis of frontotemporal dementia. J. Proteome Res. *16*, 999–1013.

Fink, J.K. (2013). Hereditary spastic paraplegia: clinico-pathologic features and emerging molecular mechanisms. Acta Neuropathol. *126*, 307–328.

Freund, A., Zhong, F.L., Venteicher, A.S., Meng, Z., Veenstra, T.D., Frydman, J., and Artandi, S.E. (2014). Proteostatic control of telomerase function through TRiC-mediated folding of TCAB1. Cell *159*, 1389–1403.

Gabrych, D.R., Lau, V.Z., Niwa, S., and Silverman, M.A. (2019). Going too far is the same as falling short(dagger): kinesin-3 family members in hereditary spastic paraplegia. Front. Cell. Neurosci. *13*, 419.

Harding, A.E. (1983). Classification of the hereditary ataxias and paraplegias. Lancet *1*, 1151–1155.

Helbig, K.L., Hedrich, U.B., Shinde, D.N., Krey, I., Teichmann, A.C., Hentschel, J., Schubert, J., Chamberlin, A.C., Huether, R., Lu, H.M., et al. (2016). A recurrent mutation in KCNA2 as a novel cause of hereditary spastic paraplegia and ataxia. Ann. Neurol. *80*.

Huttlin, E.L., Bruckner, R.J., Paulo, J.A., Cannon, J.R., Ting, L., Baltier, K., Colby, G., Gebreab, F., Gygi, M.P., Parzen, H., et al. (2017). Architecture of the human interactome defines protein communities and disease networks. Nature *545*, 505–509.

Ilgaz Aydinlar, E., Rolfs, A., Serteser, M., and Parman, Y. (2014). Mutation in FAM134B causing hereditary sensory neuropathy with spasticity in a Turkish family. Muscle Nerve *49*, 774–775.

Jassal, B., Matthews, L., Viteri, G., Gong, C., Lorente, P., Fabregat, A., Sidiropoulos, K., Cook, J., Gillespie, M., Haw, R., et al. (2020). The reactome pathway knowledgebase. Nucleic Acids Res. *48*, D498–D503.

Koh, G.C., Porras, P., Aranda, B., Hermjakob, H., and Orchard, S.E. (2012). Analyzing protein-protein interaction networks. J. Proteome Res. *11*, 2014–2031.

Kropatsch, R., Schmidt, H.M., Buttkereit, P., Epplen, J.T., and Hoffjan, S. (2019). BICD2 mutational analysis in hereditary spastic paraplegia and hereditary motor and sensory neuropathy. Muscle Nerve *59*, 484–486.

Lee, C.A., and Blackstone, C. (2020). ER morphology and endo-lysosomal crosstalk: functions and disease implications. Biochim. Biophys. Acta Mol. Cell Biol. Lipids *1865*, 158544.

Leong, W.F., and Chow, V.T. (2006). Transcriptomic and proteomic analyses of rhabdomyosarcoma cells reveal differential cellular gene expression in response to enterovirus 71 infection. Cell. Microbiol. *8*, 565–580.

Liao, Y.-H., Hsu, S.-M., and Huang, P.-H. (2007). ARMS depletion facilitates UV irradiation–induced apoptotic cell death in melanoma. Cancer Res. *67*, 11547–11556.

Licata, L., Briganti, L., Peluso, D., Perfetto, L., Iannuccelli, M., Galeota, E., Sacco, F., Palma, A., Nardozza, A.P., Santonico, E., et al. (2012). MINT, the molecular interaction database: 2012 update. Nucleic Acids Res. *40*, D857–D861.

Liu, N., Chen, J., Xu, C., Shi, T., and Li, J. (2019). Hereditary spastic paraplegia associated with a rare IFIH1 mutation: a case report and literature review. Hereditas *156*, 28.

Lo Giudice, T., Lombardi, F., Santorelli, F.M., Kawarai, T., and Orlacchio, A. (2014). Hereditary spastic paraplegia: clinical-genetic characteristics and evolving molecular mechanisms. Exp. Neurol. *261*, 518–539.

Lorrain, M. (1898). Contribution à l'étude de la paraplégie spasmodique familiale: travail de la clinique des maladies du système nerveux à la Salpêtrière (G. Steinheil)).

Luck, K., Kim, D.K., Lambourne, L., Spirohn, K., Begg, B.E., Bian, W., Brignall, R., Cafarelli, T., Campos-Laborie, F.J., Charloteaux, B., et al. (2020). A reference map of the human binary protein interactome. Nature *580*, 402–408.

Manzoni, C., Lewis, P.A., and Ferrari, R. (2020). Network analysis for complex neurodegenerative diseases. Curr. Genet. Med. Rep. *8*, 17–25.

Mi, H., Huang, X., Muruganujan, A., Tang, H., Mills, C., Kang, D., and Thomas, P.D. (2017). PANTHER version 11: expanded annotation data from Gene Ontology and Reactome pathways, and data analysis tool enhancements. Nucleic Acids Res. *45*, D183–D189.

Novarino, G., Fenstermaker, A.G., Zaki, M.S., Hofree, M., Silhavy, J.L., Heiberg, A.D., Abdellateef, M., Rosti, B., Scott, E., Mansour, L., et al. (2014). Exome sequencing links corticospinal motor neuron disease to common neurodegenerative disorders. Science *343*, 506–511.

Oliver, S. (2000). Guilt-by-association goes global. Nature *403*, 601–603.

Orchard, S., Ammari, M., Aranda, B., Breuza, L., Briganti, L., Broackes-Carter, F., Campbell, N.H., Chavali, G., Chen, C., Del-Toro, N., et al. (2014). The MIntAct project–IntAct as a common curation platform for 11 molecular interaction databases. Nucleic Acids Res. *42*, D358–D363.

Orchard, S., Kerrien, S., Abbani, S., Aranda, B., Bhate, J., Bidwell, S., Bridge, A., Briganti, L., Brinkman, F.S., Cesareni, G., et al. (2012). Protein interaction data curation: the International Molecular Exchange (IMEx) consortium. Nat. Methods *9*, 345–350.

Oughtred, R., Stark, C., Breitkreutz, B.J., Rust, J., Boucher, L., Chang, C., Kolas, N., O'donnell, L., Leung, G., Mcadam, R., et al. (2019). The BioGRID interaction database: 2019 update. Nucleic Acids Res. *47*, D529–D541.

Parodi, L., Coarelli, G., Stevanin, G., Brice, A., and Durr, A. (2018). Hereditary ataxias and paraparesias: clinical and genetic update. Curr. Opin. Neurol. *31*, 462–471.

Reimand, J., Arak, T., Adler, P., Kolberg, L., Reisberg, S., Peterson, H., and Vilo, J. (2016). g:Profiler-a web server for functional interpretation of gene lists (2016 update). Nucleic Acids Res. *44*, W83–W89.

Rolland, T., Tasan, M., Charloteaux, B., Pevzner, S.J., Zhong, Q., Sahni, N., Yi, S., Lemmens, I., Fontanillo, C., Mosca, R., et al. (2014). A proteome-scale map of the human interactome network. Cell *159*, 1212–1226.

Rousseeuw, P.J. (1987). Silhouettes: a graphical aid to the interpretation and validation of cluster analysis. J. Comput. Appl. Math. *13*, 53–65.

Schaefer, M.H., Serrano, L., and Andrade-Navarro, M.A. (2015). Correcting for the study bias associated with protein-protein interaction measurements reveals differences between protein degree distributions from different cancer types. Front. Genet. *6*, 260.

Shimazaki, H., Honda, J., Naoi, T., Namekawa, M., Nakano, I., Yazaki, M., Nakamura, K., Yoshida, K., Ikeda, S., Ishiura, H., et al. (2014). Autosomal-recessive complicated spastic paraplegia with a novel lysosomal trafficking regulator gene mutation. J. Neurol. Neurosurg. Psychiatry *85*, 1024–1028.

Simone, M., Trabacca, A., Panzeri, E., Losito, L., Citterio, A., and Bassi, M.T. (2018). KIF5A and ALS2 variants in a family with hereditary spastic paraplegia and amyotrophic lateral sclerosis. Front. Neurol. *9*, 1078.

Spiegel, R., Pines, O., Ta-Shma, A., Burak, E., Shaag, A., Halvardson, J., Edvardson, S., Mahajna, M., Zenvirt, S., Saada, A., et al. (2012). Infantile cerebellar-retinal degeneration associated with a mutation in mitochondrial aconitase, ACO2. Am. J. Hum. Genet. *90*, 518–523.

Strümpell, A. (1880). Beiträge zur Pathologie des Rückenmarks. Arch. Psychiatr.Nervenkr. *10*, 676–717.

Suzuki, R., and Shimodaira, H. (2006). Pvclust: an R package for assessing the uncertainty in hierarchical clustering. Bioinformatics *22*, 1540–1542.

Synofzik, M., and Schule, R. (2017). Overcoming the divide between ataxias and spastic paraplegias: shared phenotypes, genes, and pathways. Mov Disord. *32*, 332–345.

The Gene Ontology, C. (2019). The gene ontology resource: 20 years and still GOing strong. Nucleic Acids Res. *47*, D330–D338.

Tomkins, J.E., Dihanich, S., Beilina, A., Ferrari, R., Ilacqua, N., Cookson, M.R., Lewis, P.A., and Manzoni, C. (2018). Comparative protein interaction network analysis identifies shared and distinct functions for the human ROCO proteins. Proteomics *18*, e1700444.

Tomkins, J.E., Ferrari, R., Vavouraki, N., Hardy, J., Lovering, R.C., Lewis, P.A., Mcguffin, L.J., and Manzoni, C. (2020). PINOT: an intuitive resource for integrating protein-protein interactions. Cell Commun. Signal. *18*, 92.

UniProt, C. (2019). UniProt: a worldwide hub of protein knowledge. Nucleic Acids Res. *47*, D506–D515.

Verny, C., Guegen, N., Desquiret, V., Chevrollier, A., Prundean, A., Dubas, F., Cassereau, J., Ferre, M., Amati-Bonneau, P., Bonneau, D., et al. (2011). Hereditary spastic paraplegia-like disorder due to a mitochondrial ATP6 gene point mutation. Mitochondrion *11*, 70–75.

Wagner, M., Osborn, D.P.S., Gehweiler, I., Nagel, M., Ulmer, U., Bakhtiari, S., Amouri, R., Boostani, R., Hentati, F., Hockley, M.M., et al. (2019). Bi-allelic variants in RNF170 are associated with hereditary spastic paraplegia. Nat. Commun. *10*, 4790.

Wang, J., Vasaikar, S., Shi, Z., Greer, M., and Zhang, B. (2017). WebGestalt 2017: a more comprehensive, powerful, flexible and interactive gene set enrichment analysis toolkit. Nucleic Acids Res. *45*, W130–W137.

Wewer Albrechtsen, N.J., Geyer, P.E., Doll, S., Treit, P.V., Bojsen-Moller, K.N., Martinussen, C., Jorgensen, N.B., Torekov, S.S., Meier, F., Niu, L., et al. (2018). Plasma proteome profiling reveals dynamics of inflammatory and lipid homeostasis markers after roux-en-Y gastric bypass surgery. Cell Syst. *7*, 601–612.e3.

Zhao, M., Chen, Y.J., Wang, M.W., Lin, X.H., Dong, E.L., Chen, W.J., Wang, N., and Lin, X. (2019). Genetic and clinical profile of Chinese patients with autosomal dominant spastic paraplegia. Mol. Diagn. Ther. *23*, 781–789.

STAR★METHODS

KEY RESOURCES TABLE

REAGENT or RESOURCE	SOURCE	IDENTIFIER
Deposited data		
Neuromuscular disease center database	Washington University in St. Louis; http://neuromuscular.wustl.edu/	RRID:SCR_007305
PINOT	Bioinformatic web server of University of Reading; http://www.reading.ac.uk/bioinf/PINOT/PINOT_form.html	N/A
Software and algorithms		
R	R Project for Statistical Computing; http://www.r-project.org/	RRID:SCR_001905
Cytoscape	Institute for Systems Biology; Washington; USA , University of California at San Diego; California; USA; http://cytoscape.org	RRID:SCR_003032
gProfiler	BIIT - Bioinformatics Algorithmics and Data Mining Group; http://biit.cs.ut.ee/gprofiler/	RRID:SCR_006809
Panther	University of Southern California; Los Angeles; USA; http://www.pantherdb.org/	RRID:SCR_004869
WebGestalt	Vanderbilt University; Tennessee; USA; http://www.webgestalt.org/	RRID:SCR_006786

RESOURCE AVAILABILITY

Lead contact

Further information and requests for resources and protocols should be directed to and will be fulfilled by the lead contact, Dr Claudia Manzoni (c.manzoni@ucl.ac.uk).

Materials availability

This study did not generate new unique reagents.

Data and code availability

The published article includes all datasets and code generated or analyzed during this study. The main resource used in this study was PINOT, whose code is freely available to download from the help-page of the tool: http://www.reading.ac.uk/bioinf/PINOT/PINOT_help.html#select.

METHOD DETAILS

Selection of seeds

The protein products of 83 genes were selected as seeds based on their clinical relevance for HSPs (de Souza et al., 2017), among which 17 have not been widely recognised as HSP genes hereafter referred to as test seeds. The list of HSP seeds (n=66) is: ALDH18A1, AMPD2, AP4B1, AP4E1, AP4M1, AP4S1, AP5Z1, ARL6IP1, ARSI, ATL1, ATP13A2, B4GALNT1, BSCL2, C12orf65, C19orf12, CAPN1, CPT1C, CYP2U1, CYP7B1, DDHD1, DDHD2, DSTYK, ENTPD1, ERLIN1, ERLIN2, FA2H, FARS2, GBA2, GJC2, HSPD1, IBA57, KIF1A, KIF1C, KIF5A, KLC2, L1CAM, MAG, MARS, NIPA1, NT5C2, PGAP1, PLP1, PNPLA6, RAB3GAP2, REEP1, REEP2, RTN2, SLC16A2, SLC33A1, SPART, SPAST, SPG7, SPG11, SPG21, TECPR2, TFG, TPP1, UBAP1, UCHL1, USP8, VPS37A, WASHC5, WDR48, ZFR, ZFYVE26 and ZFYVE27.

The list of HSP test seeds (n=17) is: ACO2 (Bouwkamp et al., 2018), ALS2 (Simone et al., 2018), BICD2 (Kropatsch et al., 2019), CCDC50, CCT5 (Bouhouche et al., 2006), EXOSC3 (Blackstone, 2018a), GAD1 (Lo Giudice et al., 2014), HACE1 (Akawi et al., 2015), IFIH1 (Liu et al., 2019), KCNA2 (Helbig et al., 2016), KIDINS220 (Zhao et al., 2019), LYST (Shimazaki et al., 2014), MT-ATP6 (Verny et al., 2011), MT-CO3 (Blackstone, 2018b), MT-ND4 (Clarencon et al., 2006), RETREG1 (Ilgaz Aydinlar et al., 2014) and SELENOI (Ahmed et al., 2017)

Collection of PPIs and HSP-PPINs

The 83 seeds were used as the input to query the PINOT webtool (Tomkins et al., 2020) [http://www.reading.ac.uk/bioinf/PINOT/PINOT_form.html]. PINOT produces a list of experimentally demonstrated binary PPIs containing unique, human PPI data obtained by merging and processing PPI data from 7

databases: BioGrid (Oughtred et al., 2019), InnateDB (Breuer et al., 2013), IntAct (Orchard et al., 2014), MBInfo [https://www.mechanobio.info/], MINT (Licata et al., 2012), UniProt (UniProt, 2019) and bhf-ucl.

Through PINOT, interactions are filtered and scored based on the number of publications that report a particular interaction and the number of different methods used for their detection. The interactions provided from PINOT were then screened to remove PPIs with a final score <3 (those interactions without replication in the curated literature). The retained interactions were visualised using Cytoscape (RRID: SCR_003032; v3.7.2), thus creating the global HSP-PPIN.

Each node in the network was scored based on the number of seeds to which it connected. The nodes interacting with more than one seed, referred to as "inter-interactomes hubs (IIHs)" (Ferrari et al., 2017), were used to extract a subnetwork composed of IIHs and the connected seeds. This subnetwork was termed the "core" HSP-PPIN.

The interactions for the global HSP and core HSP networks were downloaded on the 09/07/2019, PINOT (beta version), using the stringent and *Homo sapiens* filters (default).

Enrichment analyses

The subset of proteins composing the core HSP network underwent enrichment analysis (Biological Processes [BPs] and/or Cellular Components [CCs] Gene Ontology [GO] annotations). The consistency of the results was evaluated by using 3 independent online tools, which utilise different algorithms, multiple test correction and/or versions of the GO database. In particular: g:Profiler (RRID: SCR_006809; July 2019, Over-representation enrichment analysis (Fisher's one tailed test), Bonferroni's corrections, GO database release 11/07/2019, excluding electronic annotations and analysed against the annotated human genome) (Reimand et al., 2016) [https://biit.cs.ut.ee/gprofiler/gost], Gene Ontology using Panther's tool (RRID: SCR_004869; September/October 2019, Binomial test, Bonferroni's corrections, GO database release 03/07/2019, analysed against the human genome) (Ashburner et al., 2000; The Gene Ontology, 2019; Mi et al., 2017) [http://geneontology.org/ and http://pantherdb.org/] and WebGestalt (WEB-based GEne SeT AnaLysis Toolkit, RRID: SCR_006786; October 2019, Over-representation enrichment analysis (Hypergeometric test), FDR, GO database release 14/01/2019, analysed against the protein coding human genome) (Wang et al., 2017) [http://www.webgestalt.org/].

The output of the functional enrichment includes a list of enriched GO terms and their respective enrichment ratio which can be calculated using the following formulas:

$$Enrichment\ Ratio = \frac{\mathrm{Ng}}{\mathrm{Nexp\_g}} \quad \text{(Equation 1)}$$

$$Nexp\_g = \frac{\mathrm{Ntg} * \mathrm{NgGO}}{\mathrm{Ntag}} \quad \text{(Equation 2)}$$

where Ng is the number of genes with a GO term in the data, Nexp_g the number of expected genes with a GO term in the data, Ntg the number of genes in the data, NgGO the number of genes annotated with a GO term in the GO database, and Ntag the total number of annotated genes in the GO database.

The enriched BP and CC GO terms were grouped by semantic similarity into semantic classes using in-house developed dictionaries. The semantic classes were further clustered into functional blocks and location blocks, respectively. The GO terms classified in the semantic classes "general" and "metabolism" were not included in the analysis as they refer to GO terms that provide limited functional specificity to the analysis (Ferrari et al., 2017).

Finally, in order to reduce any tool specific bias, only the functional or location blocks confirmed to be enriched by at least 2 of the 3 enrichment tools (g:Profiler, PantherGO and WebGestalt) were retained for further analysis. Particularly, for those blocks that were replicated across at least 2 tools, we analysed the merge of their semantic classes resulting from each individual tool. Additionally, only the terms that were enriched in association with at least 4 genes were retained.

The comparison of the clusters' enrichment profiles for BP and CC was performed by calculating the following ratio for each block:

$$\frac{\%cluster - \%core}{\%core} \quad \text{(Equation 3)}$$

where %cluster is the percentage of GO terms of a cluster, and %core is the percentage of GO terms of the core-HSP-PPIN.

In the case that the aforementioned ratio of the functional or location block had the value of zero for the core dataset, since dividing by zero results to ∞, we set up 25 as the maximum value and -25 as the minimum value for visualisation purposes.

Pathway enrichment was performed using Reactome's online analysis tool (RRID: SCR_003485; v69 & v70 in September and December 2019) (Jassal et al., 2020) [https://reactome.org/PathwayBrowser/#TOOL=AT]. The pathways that were significantly enriched (p-value<0.05) were retained and filtered further to remove those with 3 or less proteins involved.

The associations of HSP genes with clinical phenotypes were collected from the Neuromuscular Disease Center database, (RRID: SCR_007305; https://neuromuscular.wustl.edu/spinal/fsp.html [Accessed 29/04/2020 2020]).

Text mining was performed on the GO-BP terms after the merging of results from the 3 tools. The number of terms related to axons, cytoskeleton, endosomes, membranes, neurons, projections and vesicles were counted based on the presence of "axo*", "cytoskelet*", "endos*", "membrane*" "microtubu*", "vesic*", "neuro*" and "projections*", respectively. An enrichment analysis was performed using the same key words, based on their frequency in the results versus in the in-house dictionary that included a collection of GO terms, using the described Equations (1) and (2).

PCA & Hierarchical clustering

In order to compare functional enrichment profiles, Principal Component Analysis (PCA) was conducted through R (R Project for Statistical Computing, RRID: SCR_001905; v. 4.0.2) using the prcomp() function of the stats package. The analysis of the number and percentage of GO terms in each functional block were both rendered necessary due to the substantial difference in the number of resulting GO terms of the 6 groups, whose functional enrichment profiles were compared (22<n<114) (Table S4).

Hierarchical clustering was performed using the hclust() function (R stats package) for the groups in the PCA plot, using Euclidean as a distance measure for row clustering. However, one unit of distance in the x axis of the PCA plot is more important than on the y axis, due to PC1 (x axis) explaining more variation than PC2 (y axis) (63% and 25.8%, respectively for the analysis based on the percentage of GO terms). Thus, the coordinates of each point had to be transformed; they were multiplied by the explained variation, so that the distance between points can have the same significance in any direction and can thus be used for hierarchical clustering. Through Hierarchical clustering, the cluster dendrogram was produced. Choosing the best fit for the number of clusters derived from Hierarchical clustering was based on the Silhouette method (Rousseeuw, 1987) and the Multiscale bootstrap resampling method (Suzuki and Shimodaira, 2006). For the former, the index/score were calculated for 2 up to 6 clusters. The latter was based on the R package "pvclust" that assigns pvclust p-values to each branch of the dendrogram, which show the confidence of the result (the higher the value, the more confident we are of the result) (Suzuki and Shimodaira, 2006) (Data S1).

QUANTIFICATION AND STATISTICAL ANALYSIS

For the analysis of the merged semantic classes from the 3 different tools, the threshold for determining statistical significance of each GO term was decreased to p = 0.0166 (=0.05/3) to account for the multiple comparisons.

The statistical analysis of the enrichment of key words was performed by running 100,000 random simulations, where these key words were extracted from the in-house dictionary, and the pnorm() value was calculated using R.

iScience, Volume *24*

Supplemental information

Integrating protein networks and machine learning for disease stratification in the Hereditary Spastic Paraplegias

Nikoleta Vavouraki, James E. Tomkins, Eleanna Kara, Henry Houlden, John Hardy, Marcus J. Tindall, Patrick A. Lewis, and Claudia Manzoni

Supplementary Figures

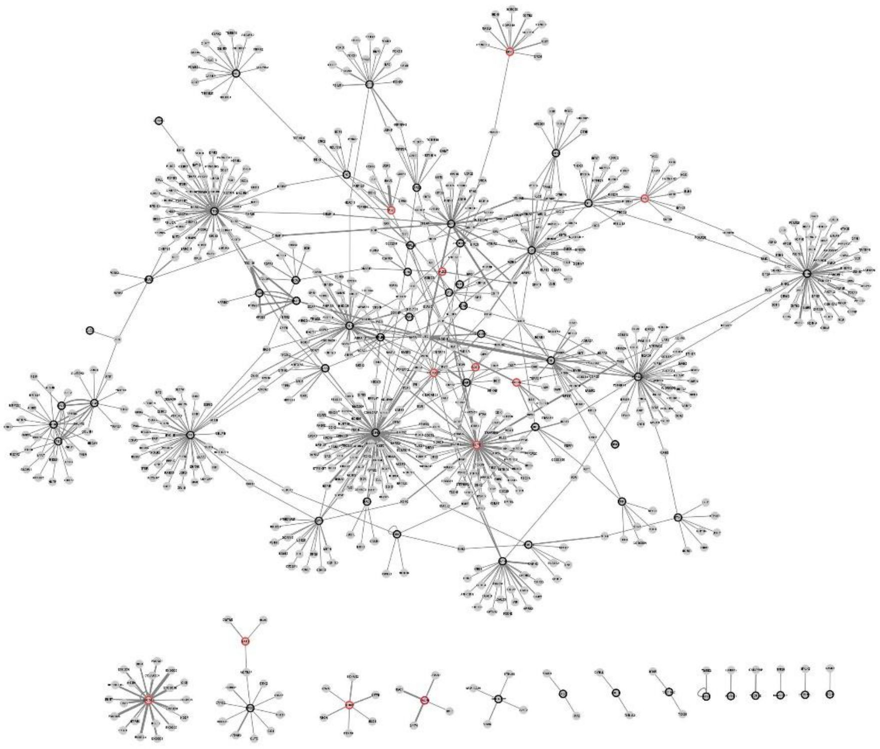

Figure S1. The global HSP-PPIN, related to Figure 1
The global HSP-PPIN is a visualisation of all binary interactions of the HSP seeds and test seeds that were collected through the online tool PINOT following filtering based on the final score. The nodes corresponding to the HSP seeds have a black border, while the test seeds have a red border. The thickness of each edge positively correlates with the final score of the respective interaction as calculated by PINOT, which acts as a proxy for interaction confidence.

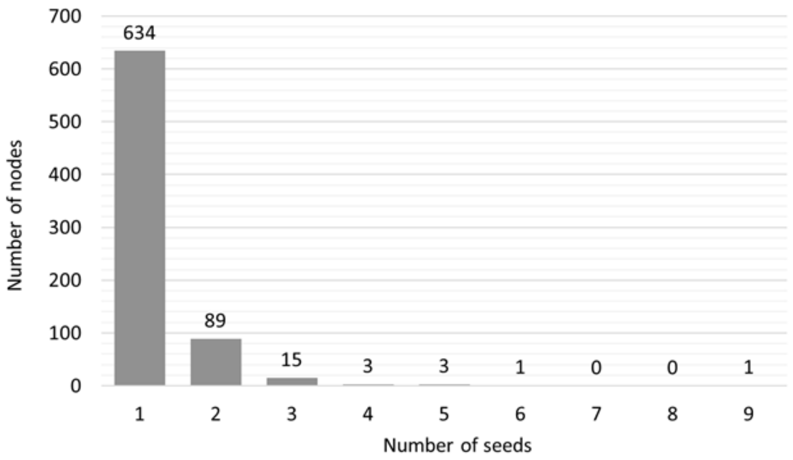

Figure S2. Node degree distribution of the proteins of the global HSP-PPIN based on their connectivity with seeds, related to Figure 1
The nodes of the global HSP-PPIN were analysed to calculate the number of seeds to which they connect. The interactors connected to one seed (n=634) were not included in further analyses.

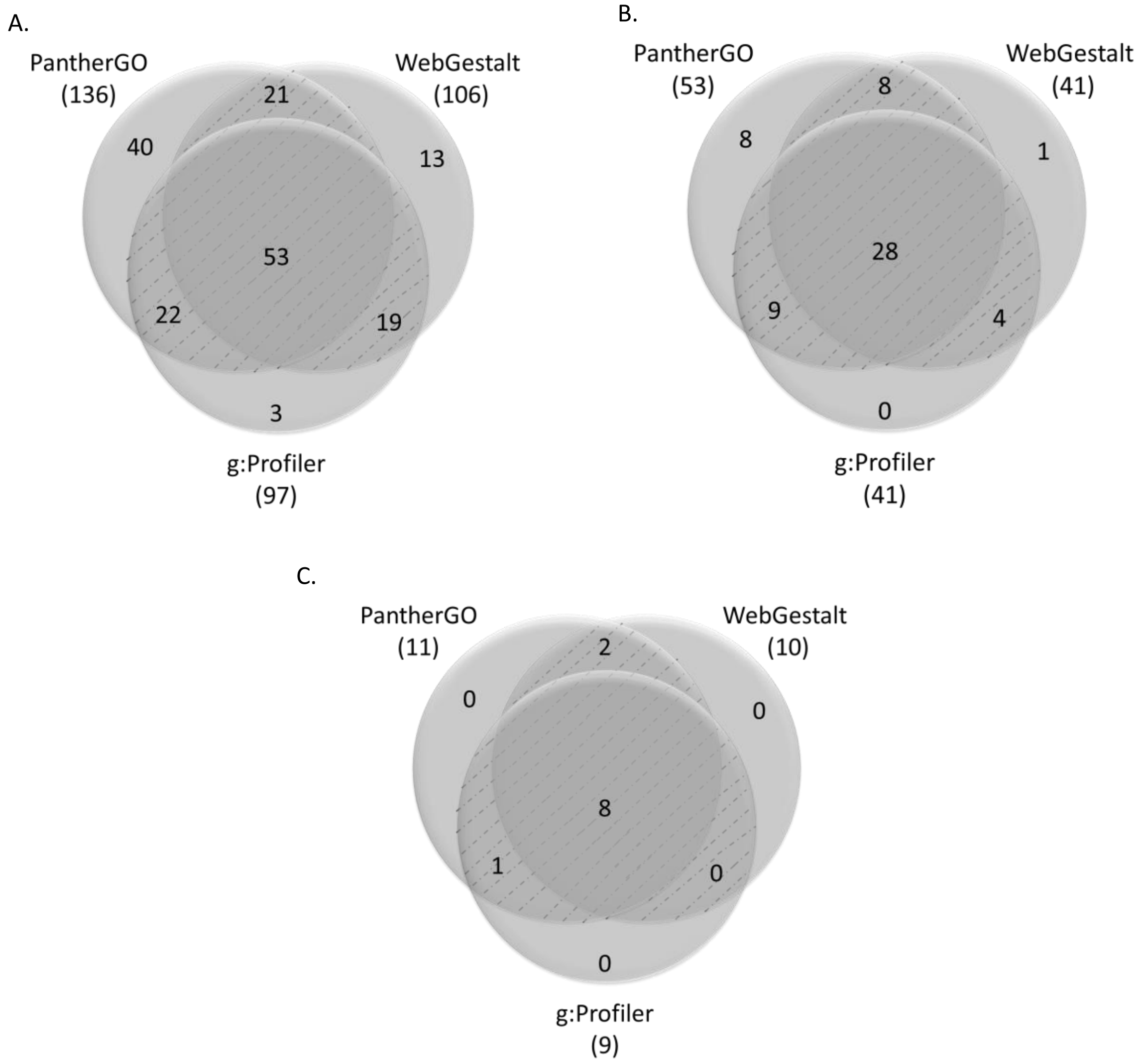

Figure S3. Overlap of the three functional enrichment tools for the analysis of the core HSP-PPIN, related to Figure 2 and 3

The results from the functional enrichment analysis of the core HSP-PPIN were compared across the functional enrichment tools used, in three levels: single GO-BP terms (n=171) (**A**), semantic classes (n=58) (**B**), and functional blocks (n=11) (**C**).

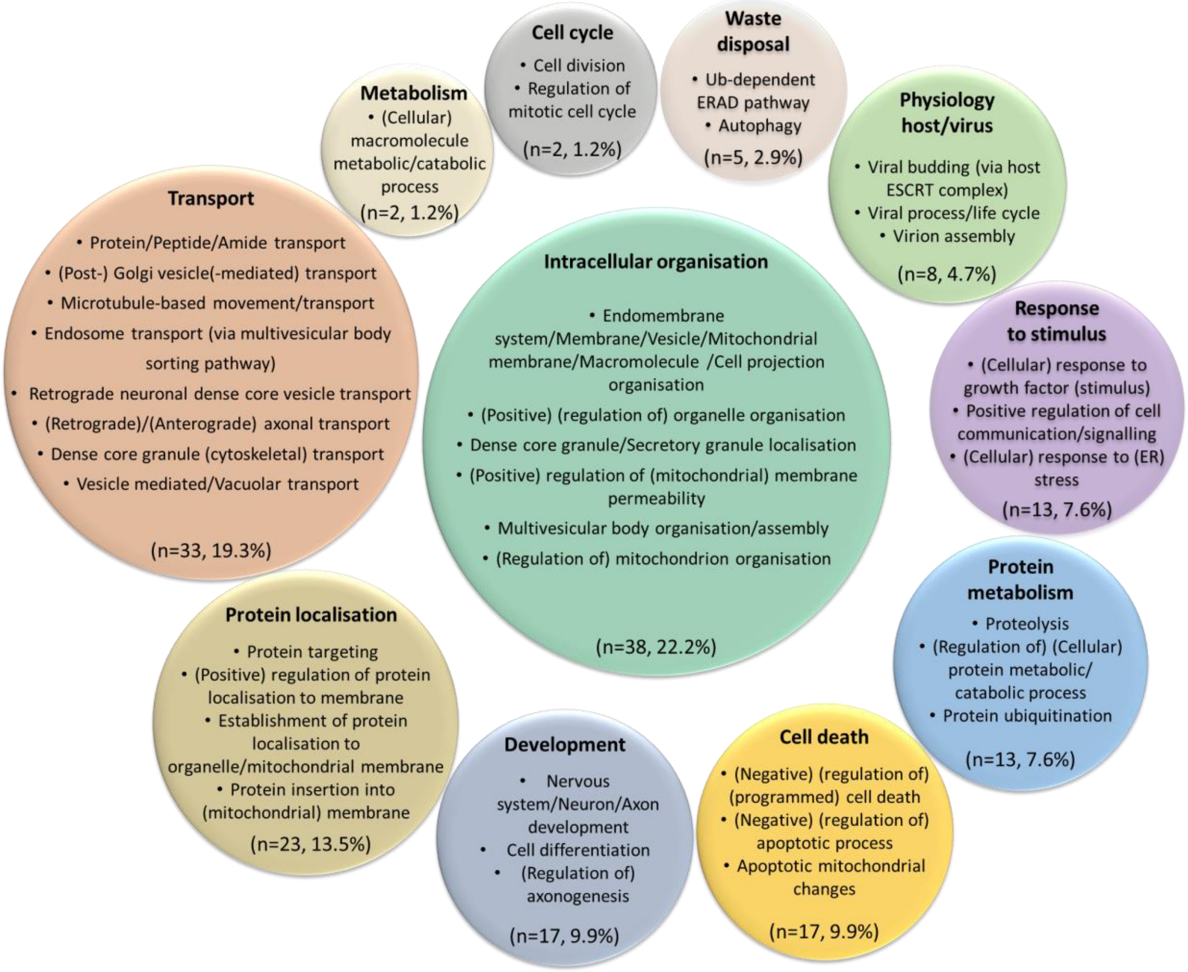

Figure S4. Detailed graphical representation of the functional enrichment of the core HSP-PPIN, related to Figure 3

Functional enrichment was performed for the components of the core HSP-PPIN. The resulted GO-BP terms (n=171) (see Table S3) were grouped into semantic classes and then into functional blocks (name of each circle, bolded). The number and percentage of terms in each functional block was calculated by merging the data from g:Profiler, WebGestalt, and PantherGO as described in STAR Methods. Examples of GO-BP terms are included inside the circle of each functional block.

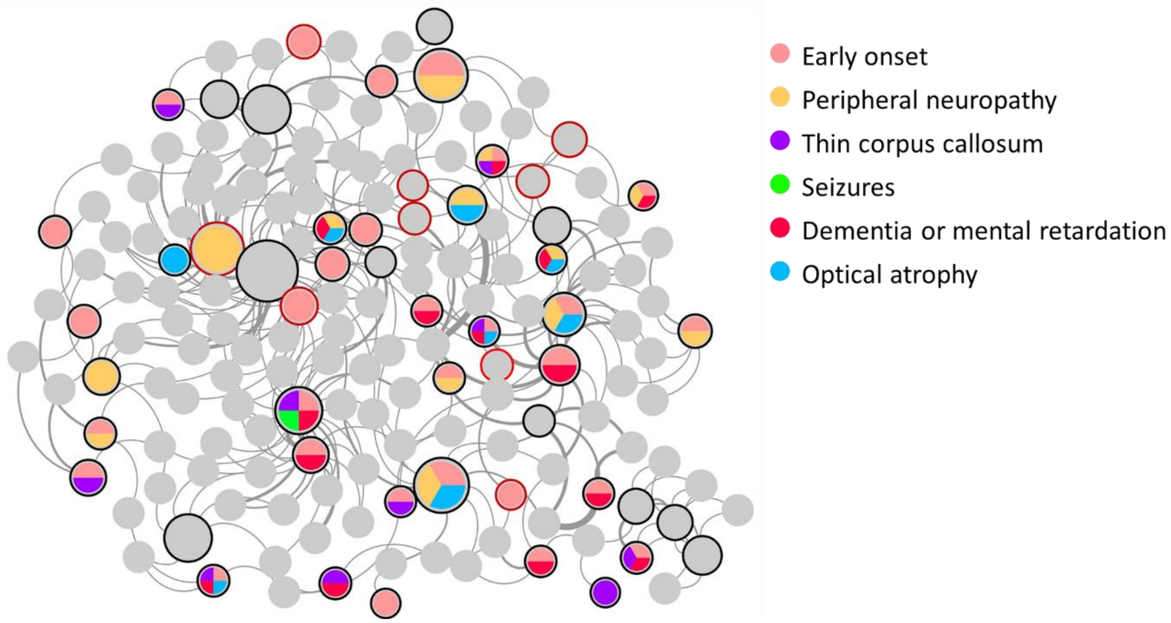

Figure S5. Visualisation of clinical characteristics of HSPs caused by each HSP gene of the core HSP-PPIN, related to Figure 4

The presence of clinical characteristics in HSPs is visualised in the core HSP-PPIN through the colour of each node. The colour-function correspondence is located on the top right of the image, while the grey nodes did not present any of the clinical features. The nodes corresponding to the HSP seeds have a black border, while the test seeds have a red border. The size of each node correlates with its degree. The thickness of each edge correlates with its final score as calculated by PINOT.

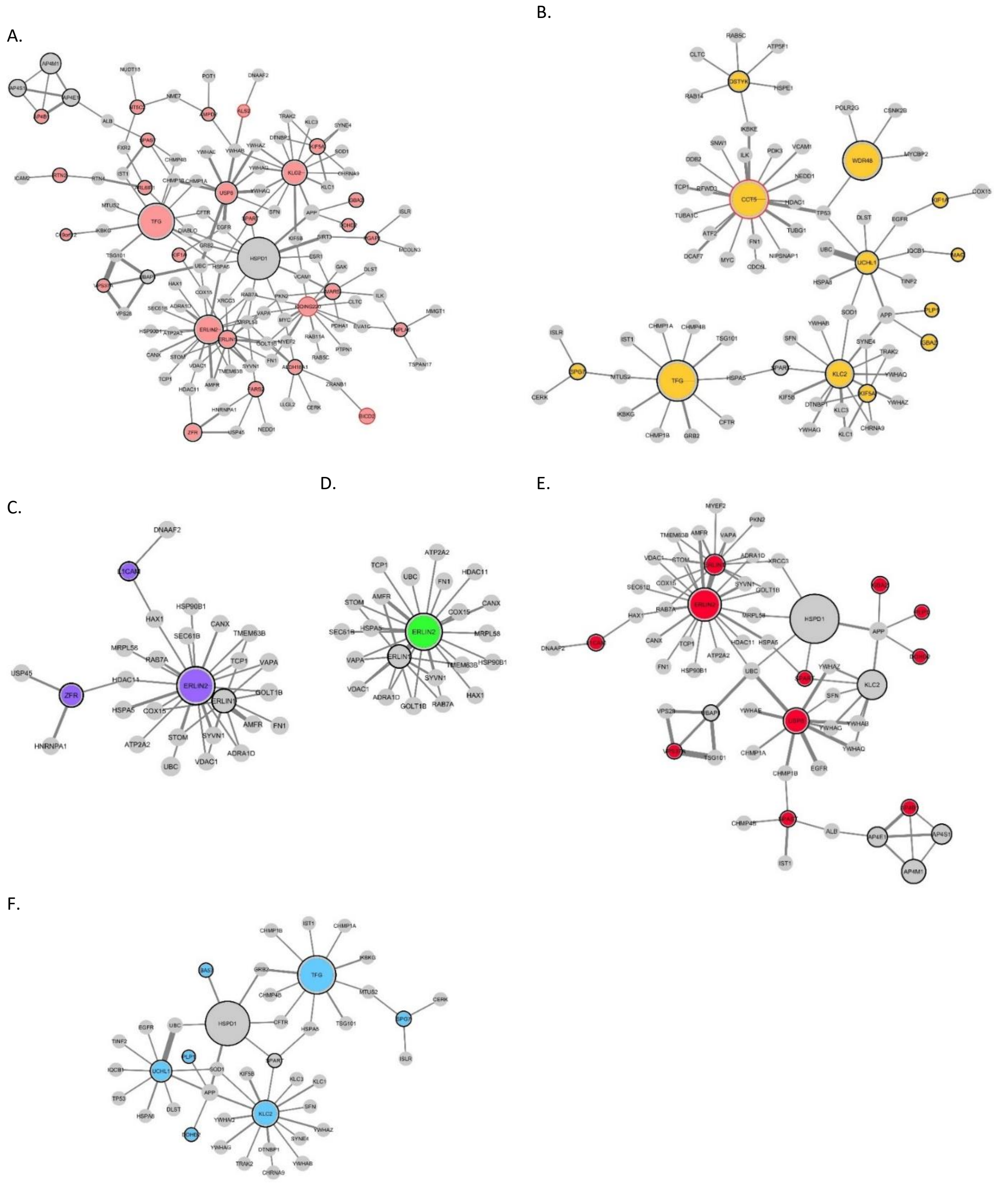

Figure S6. Comparison of the core network of seeds with the analysed clinical features, related to Figure 4

The presence of clinical characteristics in HSPs is visualised in the core networks by the colour of each node for early onset (**A**), peripheral neuropathy (**B**), thin corpus callosum (**C**), seizures, (**D**), dementia or mental retardation (**E**), and optic atrophy (**F**), while the nodes without that clinical feature are grey. The nodes corresponding to the HSP seeds have a black border, while the test seeds have a red border. The size of each node correlates with its degree. The thickness of each edge correlates with its final score as calculated by PINOT.

A.

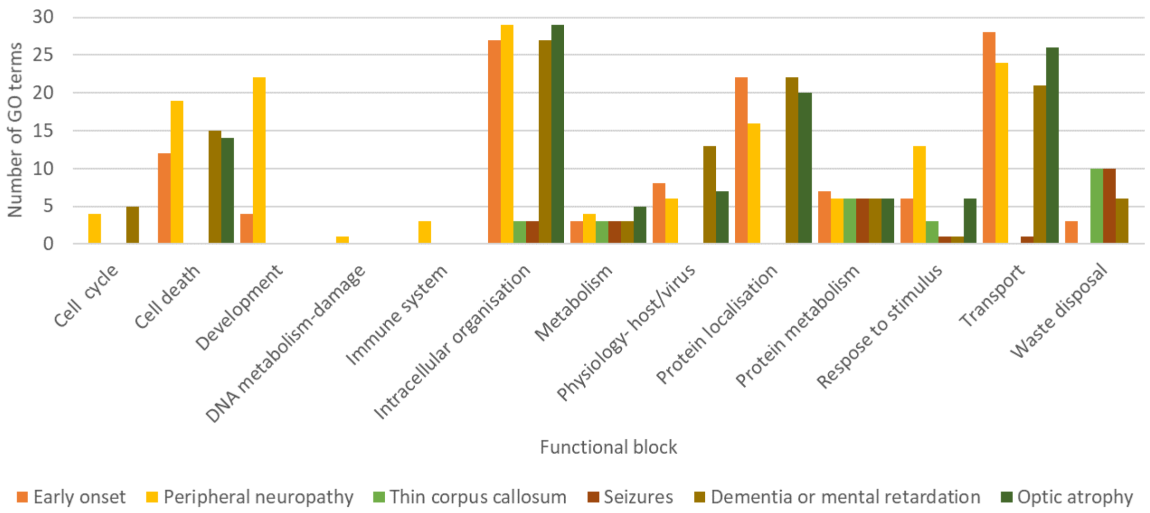

B.

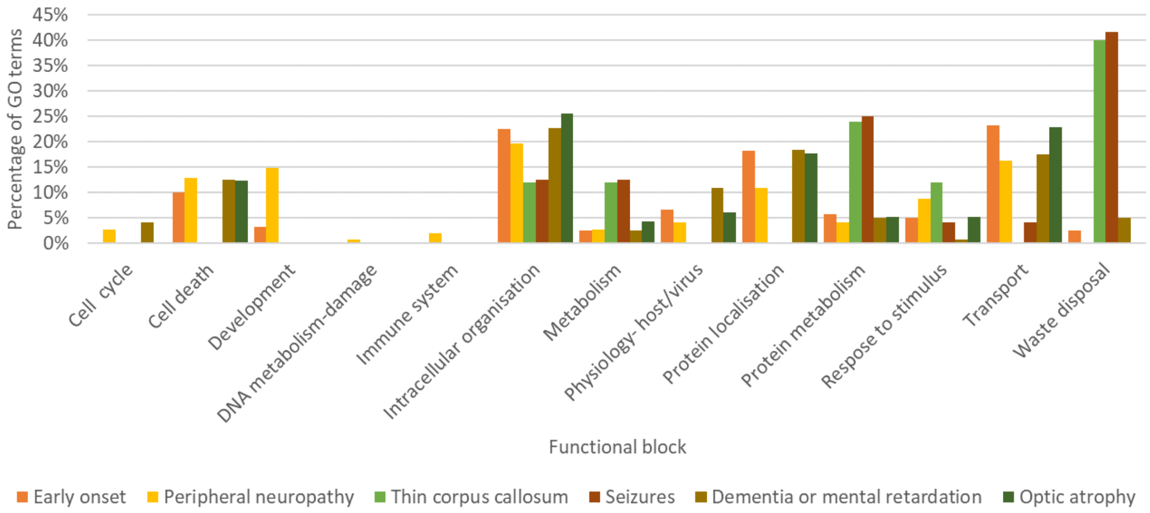

Figure S7. Functional enrichment of the core HSP-PPIN for each group related to a clinical characteristic, related to Figure 4

The number (**A**) and percentage (**B**) of the GO-BP terms in each functional block is shown for all six groups of genes related to different clinical features. The results are calculated from the functional enrichment data of g:Profiler, PantherGO, and WebGestalt (raw data in Table S4).

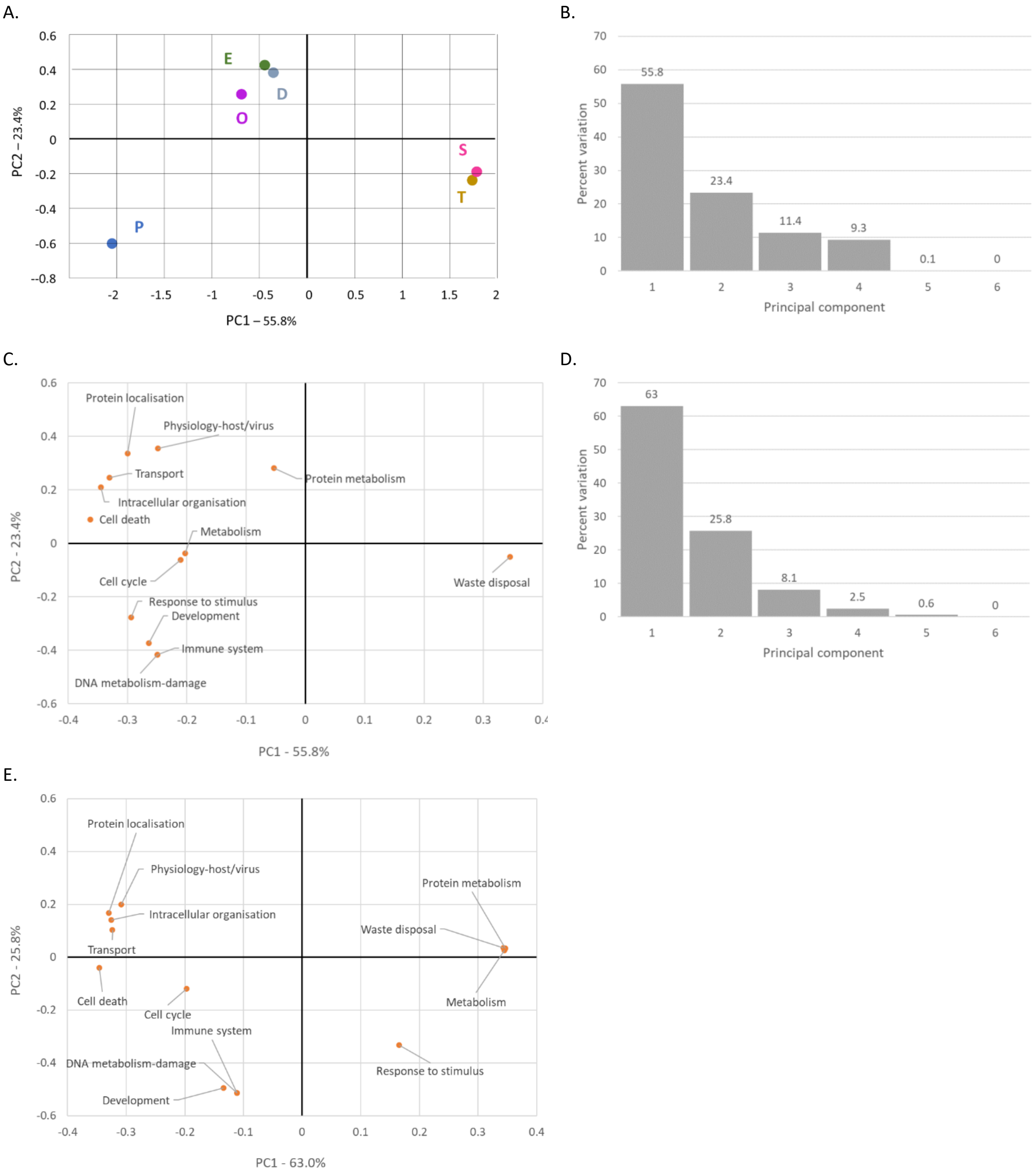

Figure S8. Comparison of the functional profiles of the 6 subdivisions of the core HSP-PPIN based on clinical features using Principal Component Analysis, related to Figure 4

The number (**A**, **B**, **C**) and percentage (**D**, **E**) of GO-BP terms for each functional block were analysed with PCA through R. (**A**) The PCA graph is showing the distribution of the gene groups in the PC1 and PC2 axes. The axes have been adjusted based on their respective explained variation. (**B**, **D**) The scree plot is showing the explained variation of the data for PC1 to PC6. (**C**, **E**) The loading scores of each variable (here functional blocks) are plotted against PC1 and PC2, indicating which functions drive the localisation of the gene groups in the PCA graph. E: Early onset, P: Peripheral neuropathy, T: Thin corpus callosum, S: Seizures, D: Dementia or mental retardation, O: Optic atrophy

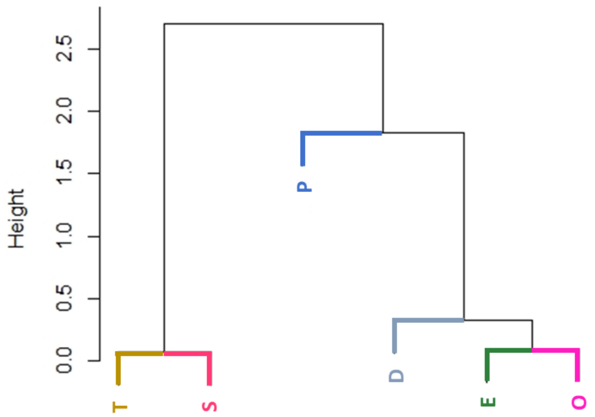

Figure S9. Cluster dendrogram for the number of GO-BP terms in the enrichment of the clinical subnetworks following PCA, related to Figure 4

Cluster dendrogram produced based on hierarchical clustering of the gene groups as analysed in Figure 4. E: Early onset, P: Peripheral neuropathy, T: Thin corpus callosum, S: Seizures, D: Dementia or mental retardation, O: Optic atrophy

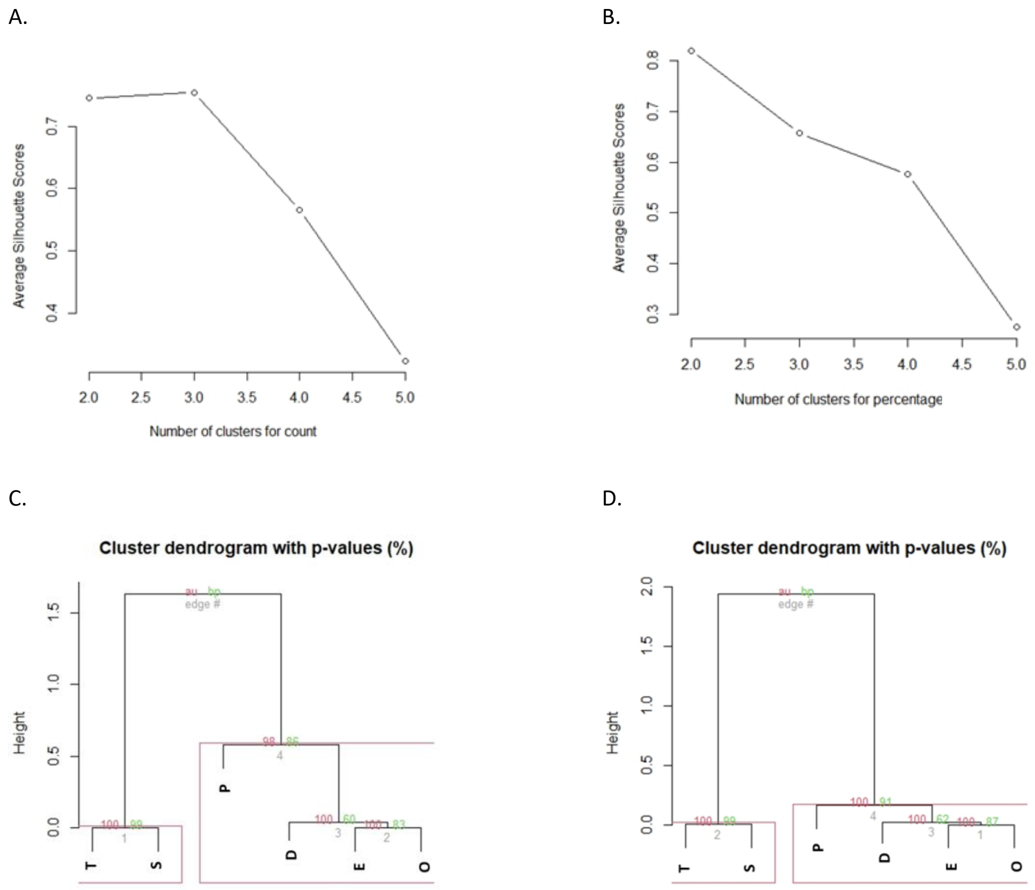

Figure S10. Evaluating the optimal number of clusters using the Silhouette method and the Multiscale bootstrap resampling through R, related to Figure 4

The analysis was based on the number (**A**, **C**) and percentage (**B**, **D**) of the GO-BP terms in functional block. The graphs based on the Silhouette method (**A**, **B**) show the optimal number of clusters being the one with the highest value, whereas based on multiscale bootstrap resampling (**C**, **D**) the recommended clusters are framed in red boxes. E: Early onset, P: Peripheral neuropathy, T: Thin corpus callosum, S: Seizures, D: Dementia or mental retardation, O: Optic atrophy

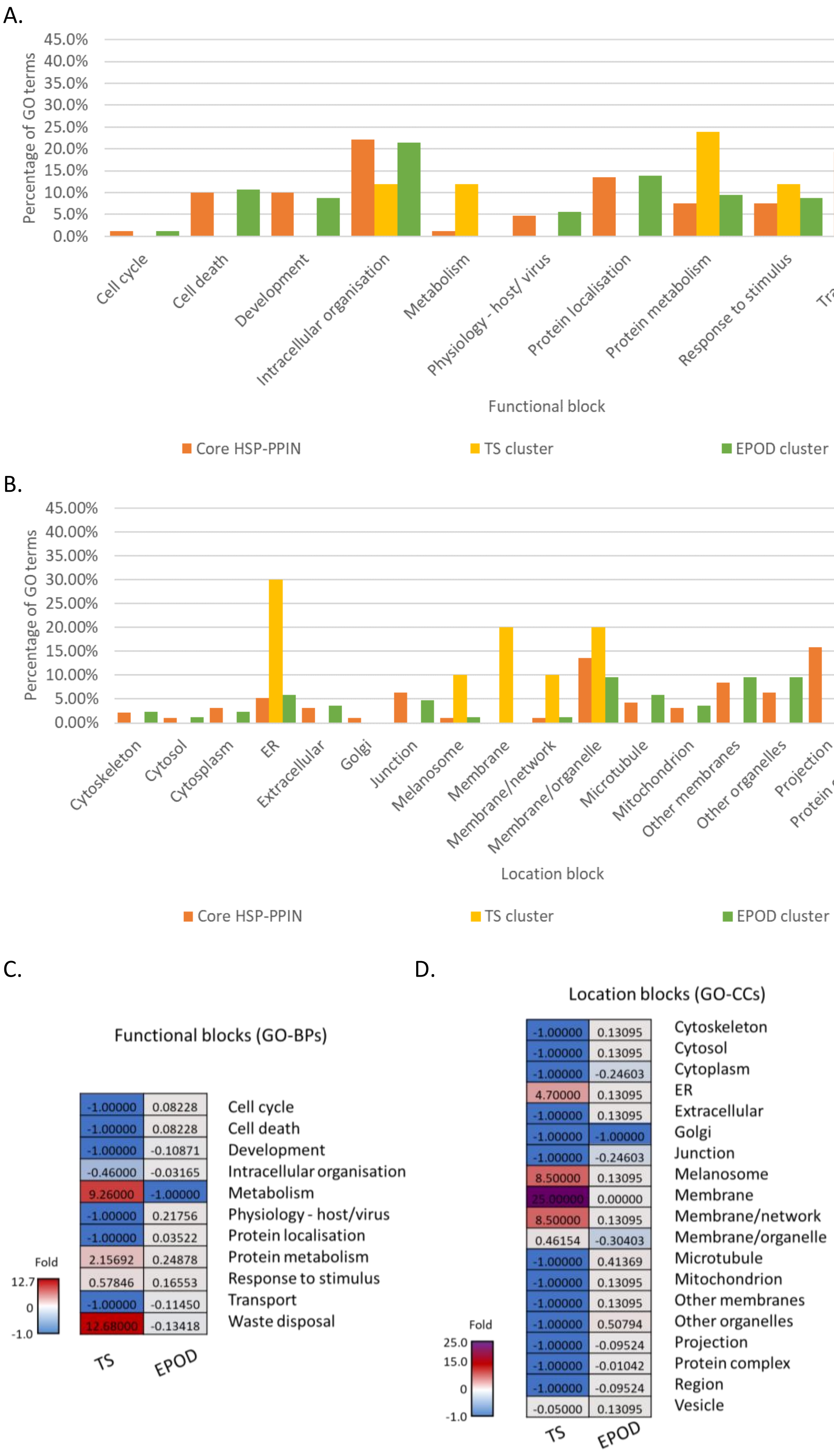
A.
Percentage of GO terms
45.0%
40.0%
35.0%
30.0%
25.0%
20.0%
15.0%
10.0%
5.0%
0.0%
Cell cycle
Cell death
Development
Intracellular organisation
Metabolism
Physiology - host/ virus
Protein localisation
Protein metabolism
Response to stimulus
Transport
Waste disposal
Functional block
Core HSP-PPIN
TS cluster
EPOD cluster
B.
Percentage of GO terms
45.00%
40.00%
35.00%
30.00%
25.00%
20.00%
15.00%
10.00%
5.00%
0.00%
Cytoskeleton
Cytosol
Cytosplasm
ER
Extracellular
Golgi
Junction
Melanosome
Membrane
Membrane/network
Membrane/organelle
Microtubule
Mitochondrion
Other membranes
Other organelles
Projection
Protein complex
Region
Vesicle
Location block
Core HSP-PPIN
TS cluster
EPOD cluster
C.
Functional blocks (GO-BPs)
-1.00000 0.08228 Cell cycle
-1.00000 0.08228 Cell death
-1.00000 -0.10871 Development
-0.46000 -0.03165 Intracellular organisation
9.26000 -1.00000 Metabolism
-1.00000 0.21756 Physiology - host/virus
-1.00000 0.03522 Protein localisation
2.15692 0.24878 Protein metabolism
0.57846 0.16553 Response to stimulus
-1.00000 -0.11450 Transport
12.68000 -0.13418 Waste disposal
Fold
12.7
0
-1.0
TS
EPOD
D.
Location blocks (GO-CCs)
-1.00000 0.13095 Cytoskeleton
-1.00000 0.13095 Cytosol
-1.00000 -0.24603 Cytoplasm
4.70000 0.13095 ER
-1.00000 0.13095 Extracellular
-1.00000 -1.00000 Golgi
-1.00000 -0.24603 Junction
8.50000 0.13095 Melanosome
25.00000 0.00000 Membrane
8.50000 0.13095 Membrane/network
0.46154 -0.30403 Membrane/organelle
-1.00000 0.41369 Microtubule
-1.00000 0.13095 Mitochondrion
-1.00000 0.13095 Other membranes
-1.00000 0.50794 Other organelles
-1.00000 -0.09524 Projection
-1.00000 -0.01042 Protein complex
-1.00000 -0.09524 Region
-0.05000 0.13095 Vesicle
Fold
25.0
15.0
0
-1.0
TS
EPOD

Figure S11. Comparison of the enrichment profile of the two clusters of the core HSP-PPIN, related to Figure 5

The analysis is based on the percentage of GO terms in each functional or location block as resulted from the enrichment of GO-BP terms (**A**, **C**) and GO-CC terms (**B**, **D**). The comparison is expressed as a fold change compared to the profile of the core HSP-PPIN (**C**, **D**).

Supplementary Tables

Table S1. Sources of variation in the clinical phenotype of HSP patients, related to Figure 1	
**Age of onset**	Early childhood up to late adulthood
**Form**	Pure and complex
**Mode of inheritance**	Autosomal recessive, autosomal dominant, X-linked, mitochondrial and unknown
**Usual Symptoms**	Bilateral spasticity and weakness of the lower body, leg hypertonicity, positive Babinski sign, muscle weakness, hyperreflexia, bladder dysfunction, loss of vibration sensation in the ankles, and pes cavus
**Additional symptoms present in some complex forms**	Cerebellar ataxia, epilepsy, cognitive or mental impairment, cataracts, retinal alteration, optic atrophy, peripheral neuropathy, dystonia, and parkinsonism

**Table S2. The gene (or locus) and protein name responsible for each HSP type, related to Figure 1**			
**Gene name (or locus)**	**Protein name (previous name)**	**UniProt identifier (SwissProt)**	**HSP type**
L1CAM	neural cell adhesion molecule L1	P32004	SPG1
PLP1	myelin proteolipid protein	P60201	SPG2
ATL1	atlastin-1	Q8WXF7	SPG3A
SPAST	spastin	Q9UBP0	SPG4
CYP7B1	25-hydroxycholesterol 7-alpha-hydroxylase	O75881	SPG5A
NIPA1	magnesium transporter NIPA1	Q7RTP0	SPG6
SPG7	paraplegin	Q9UQ90	SPG7
WASHC5	WASH complex subunit 5 (strumpellin)	Q12768	SPG8
ALDH18A1	delta-1-pyrroline-5-carboxylate synthase	P54886	SPG9A/SPG9B
KIF5A	kinesin heavy chain isoform 5A	Q12840	SPG10
SPG11	spatacsin	Q96JI7	SPG11
RTN2	reticulon-2	O75298	SPG12
HSPD1	60 kDa heat shock protein, mitochondrial	P10809	SPG13
(3q27-q28)			SPG14
ZFYVE26	zinc finger FYVE domain-containing protein 26 (spastizin)	Q68DK2	SPG15
(Xq11.2)			SPG16
BSCL2	seipin	Q96G97	SPG17
ERLIN2	erlin-2	O94905	SPG18/SPG37
(9q33-q34)			SPG19
SPART	spartin	Q8N0X7	SPG20
SPG21	maspardin	Q9NZD8	SPG21
SLC16A2	monocarboxylate transporter 8	P36021	SPG22
DSTYK	dual serine/threonine and tyrosine protein kinase	Q6XUX3	SPG23
(13q14)			SPG24
(6q23-24.1)			SPG25
B4GALNT1	beta-1,4 N-acetylgalactosaminyltransferase 1	Q00973	SPG26
(10q22.1-q24.1)			SPG27
DDHD1	phospholipase DDHD1	Q8NEL9	SPG28
(1p31.1-p21.1)			SPG29
KIF1A	kinesin-like protein KIF1A	Q12756	SPG30
REEP1	receptor expression-enhancing protein 1	Q9H902	SPG31
(14q12-q21)			SPG32

**Table S2. (continued) The gene (or locus) and protein name responsible for each HSP type, related to Figure 1**			
**Gene name (or locus)**	**Protein name (previous name)**	**UniProt identifier (SwissProt)**	**HSP type**
ZFYVE27	protrudin	Q5T4F4	SPG33
(Xq24-q25)			SPG34
FA2H	fatty acid 2-hydroxylase	Q7L5A8	SPG35
(12q23-q24)			SPG36
(4p16-p15)			SPG38
PNPLA6	neuropathy target esterase	Q8IY17	SPG39
(11p14.1-p11.2)			SPG41
SLC33A1	acetyl-coenzyme A transporter 1	O00400	SPG42
C19orf12	protein C19orf12	Q9NSK7	SPG43
GJC2	gap junction gamma-2 protein	Q5T442	SPG44
NT5C2	cytosolic purine 5'-nucleotidase	P49902	SPG45/SPG65
GBA2	non-lysosomal glucosylceramidase	Q9HCG7	SPG46
AP4B1	AP-4 complex subunit beta-1	Q9Y6B7	SPG47
AP5Z1	AP-5 complex subunit zeta-1	O43299	SPG48
TECPR2	tectonin beta-propeller repeat-containing protein 2	O15040	SPG49
AP4M1	AP-4 complex subunit mu-1	O00189	SPG50
AP4E1	AP-4 complex subunit epsilon-1	Q9UPM8	SPG51
AP4S1	AP-4 complex subunit sigma-1	Q9Y587	SPG52
VPS37A	vacuolar protein sorting-associated protein 37A	Q8NEZ2	SPG53
DDHD2	phospholipase DDHD2	O94830	SPG54
C12orf65	probable peptide chain release factor C12orf65, mitochondrial	Q9H3J6	SPG55
CYP2U1	cytochrome P450 2U1	Q7Z449	SPG56
TFG	protein TFG	Q92734	SPG57
KIF1C	kinesin-like protein KIF1C	O43896	SPG58
USP8	ubiquitin carboxyl-terminal hydrolase 8	P40818	SPG59
WDR48	WD repeat-containing protein 48	Q8TAF3	SPG60

Table S2. (continued) The gene (or locus) and protein name responsible for each HSP type, related to Figure 1			
**Gene name (or locus)**	**Protein name (previous name)**	**UniProt identifier (SwissProt)**	**HSP type**
ARL6IP1	ADP-ribosylation factor-like protein 6-interacting protein 1	Q15041	SPG61
ERLIN1	erlin-1	O75477	SPG62
AMPD2	AMP deaminase 2	Q01433	SPG63
ENTPD1	ectonucleoside triphosphate diphosphohydrolase 1	P49961	SPG64
ARSI	arylsulfatase I	Q5FYB1	SPG66
PGAP1	GPI inositol-deacylase	Q75T13	SPG67
KLC2	kinesin light chain 2	Q9H0B6	SPG68
RAB3GAP2	rab3 GTPase-activating protein non-catalytic subunit	Q9H2M9	SPG69
MARS	methionine--tRNA ligase, cytoplasmic	P56192	SPG70
ZFR	zinc finger RNA-binding protein	Q96KR1	SPG71
REEP2	receptor expression-enhancing protein 2	Q9BRK0	SPG72
CPT1C	carnitine O-palmitoyltransferase 1, brain isoform	Q8TCG5	SPG73
IBA57	putative transferase CAF17, mitochondrial	Q5T440	SPG74
MAG	myelin-associated glycoprotein	P20916	SPG75
CAPN1	calpain-1 catalytic subunit	P07384	SPG76
FARS2	phenylalanine--tRNA ligase, mitochondrial	O95363	SPG77
ATP13A2	cation-transporting ATPase 13A2	Q9NQ11	SPG78
UCHL1	ubiquitin carboxyl-terminal hydrolase isozyme L1	P09936	SPG79
UBAP1	ubiquitin-associated protein 1	Q9NZ09	SPG80
TPP1	tripeptidyl-peptidase 1	O14773	-

**Table S5. Overlap of protein composition within the TS cluster, related to Figure 4**		
	T	S
T		100.0%
S	82.1%	

**Table S6. Overlap of protein composition within the EPOD cluster, related to Figure 4**				
	E	P	D	O
E		60.3%	96.5%	81.0%
P	38.6%		36.8%	92.9%
D	48.2%	28.8%		45.2%
O	29.8%	53.4%	33.3%	

**Table S7. Overlap of protein composition between the TS and the EPOD cluster, related to Figure 4**						
	E	P	T	S	D	O
E			96.4%	100.0%		
P			17.9%	21.7%		
T	23.7%	6.8%			43.9%	4.8%
S	20.2%	6.8%			40.4%	4.8%
D			89.3%	100.0%		
O			7.1%	8.7%		
